# MACE-OFF: Short-Range Transferable Machine Learning Force Fields for Organic Molecules

**DOI:** 10.1021/jacs.4c07099

**Published:** 2025-05-19

**Authors:** Dávid Péter Kovács, J. Harry Moore, Nicholas J. Browning, Ilyes Batatia, Joshua T. Horton, Yixuan Pu, Venkat Kapil, William C. Witt, Ioan-Bogdan Magdău, Daniel J. Cole, Gábor Csányi

**Affiliations:** † Engineering Laboratory, 2152 University of Cambridge, Cambridge CB2 1PZ, U.K.; ‡ Ångström AI, 2325 Third Street, San Francisco, California 94107, United States; § 28489 Swiss National Supercomputing Centre (CSCS), 6900 Lugano, Switzerland; ∥ School of Natural and Environmental Sciences, 5994 Newcastle University, Newcastle upon Tyne NE1 7RU, U.K.; ⊥ Department of Physics and Astronomy, 154299 University College, London WC1E 6BT, U.K.; # Yusuf Hamied Department of Chemistry, 2152 University of Cambridge, Lensfield Road, Cambridge CB2 1EW, U.K.; ∇ Thomas Young Centre and London Centre for Nanotechnology, London WC1E 6BT, U.K.; ○ Department of Materials Science and Metallurgy, 2152 University of Cambridge, 27 Charles Babbage Road, Cambridge CB3 0FS, U.K.

## Abstract

Classical empirical force fields have dominated biomolecular simulations for over 50 years. Although widely used in drug discovery, crystal structure prediction, and biomolecular dynamics, they generally lack the accuracy and transferability required for first-principles predictive modeling. In this paper, we introduce MACE-OFF, a series of short-range transferable force fields for organic molecules created using state-of-the-art machine learning technology and first-principles reference data computed with a high level of quantum mechanical theory. MACE-OFF demonstrates the remarkable capabilities of short-range models by accurately predicting a wide variety of gas- and condensed-phase properties of molecular systems. It produces accurate, easy-to-converge dihedral torsion scans of unseen molecules as well as reliable descriptions of molecular crystals and liquids, including quantum nuclear effects. We further demonstrate the capabilities of MACE-OFF by determining free energy surfaces in explicit solvent as well as the folding dynamics of peptides and nanosecond simulations of a fully solvated protein. These developments enable first-principles simulations of molecular systems for the broader chemistry community at high accuracy and relatively low computational cost.

## Graphical abstract

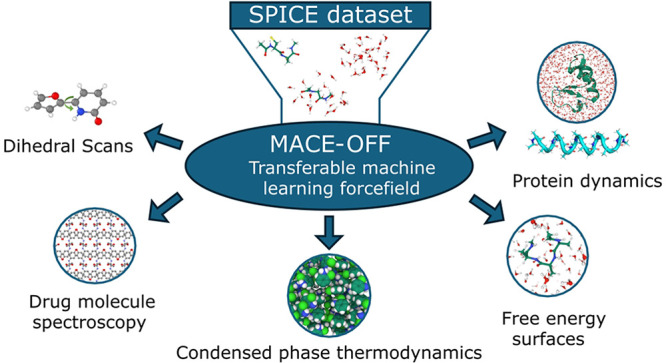

## 1 Introduction

Machine learning (ML) force fields have recently undergone major improvements in accuracy, robustness, and computational speed.^1–13^ They are now routinely used in materials chemistry contexts, where density functional theory was previously the method of choice. In these applications, available empirical force fields, such as the embedded-atom method,^14^ do not provide sufficient accuracy and transferability to describe many scientifically interesting and challenging phenomena. Successful applications of ML potentials include the simulation of quenching of amorphous silicon,^15^ determination of the phase diagrams of inorganic perovskites^16^ and alloys,^17^ and device-scale simulation of phase-change memory materials.^18^

In contrast, simulating bio-organic systems entails a different set of trade-offs, with greater emphasis on simulating large systems over long time scales. This means that empirical force fields, which sacrifice accuracy for computational speed, continue to be used routinely to study molecular liquids, crystals, biological systems, and drug-like molecules.^19–22^

Two alternatives to empirical force fields are available. The first is semiempirical quantum mechanics, such as the series of extended tight-binding models,^23^ which represents a low-cost solution for small molecules. The method is limited by its moderate accuracy compared to quantum chemistry methods, its restriction to modeling nonperiodic systems (if not in principle, but in widely used implementations), and its cubic scaling with system size.

More recently, a number of transferable machine learning force fields have also been developed for organic chemistry. By “transferable”, here, we mean that these potentials are capable of generalizing in system size and both chemical and conformation space, sufficiently well to perform stable molecular dynamics and accurate property predictions for a wide range of molecular systems beyond those on which the model was trained. The most notable are the series of ANI^24–27^ and AIMNet potentials.^8,28,29^ ANI potentials pioneered the use of local symmetry function-based feedforward neural networks^30^ trained on a large data set of organic molecular geometries^31,32^ to create transferable ML force fields. The ANI-2x model became the most widely adopted ML force field and therefore serves as one of the primary points of comparison in this paper. The ANI-2x model was recently combined with a polarizable electrostatic model^33^ in a hybrid ML/MM simulation setting and also with a neural network-based dispersion correction.^34^ The AIMNet models apply a message-passing architecture,^35^ where the initial embeddings are the ANI symmetry functions. They have also extended the applicability of the models to a larger set of chemical elements as well as to charged species. These models relax the locality assumptions by incorporating electrostatic and dispersion interactions. The PhysNet model uses a message-passing architecture,^35^ and in addition to semilocal terms, also includes long-range electrostatic and dispersion interactions.^36^

Other recent bio-organic force fields include the FENNIX model, which combines a local equivariant machine learning model with a physical long-range functional form for electrostatics and dispersion.^37^ The model was trained to reproduce the CCSD­(T)/CBS energies of small molecules and molecular dimers. It was shown that FENNIX can be used to run stable dynamics of liquid water, solvated alanine dipeptides, and an entire protein in the gas phase. However, further benchmarking of this model is required to assess the accuracy of the intramolecular potential outside the training set and of condensed-phase molecular dynamics simulations.

Similarly, the ANA2B potential employs a short-ranged ML potential, with long-ranged, classical multipolar electrostatics, polarization, and dispersion interactions.^38^ Although this long-ranged model shows promising accuracy for condensed-phase properties and crystal structure ranking, its accuracy and computational performance have not yet been demonstrated for larger biomolecules.

The GEMS model,^39^ built on the SpookyNet architecture,^40^ is another recent ML force field for biomolecular simulations. While a transferable version of this potential is capable of producing stable dynamics for challenging biomolecular systems, certain properties, for example, the folding dynamics of small peptides, require the addition of system-specific training configurations in order to accurately reproduce experimental properties. More recently, the transferable SO3LR potential was introduced by the same group of researchers.^41^ This is based on the equivariant SO3krates architecture and also includes analytic dispersion and electrostatic long-range contributions. While this potential is capable of fast and stable simulations of biomolecular systems, we are not yet aware of more extensive quantitative benchmarking on off-equilibrium energetics or thermodynamic properties.

In this paper, we introduce a family of purely local (i.e., short-range), transferable bio-organic machine learning force fields, which we refer to collectively as MACE-OFF. The force fields are parametrized for the 10 most important chemical elements for organic chemistry: H, C, N, O, F, P, S, Cl, Br, and I. They are capable of accurately describing intra- and intermolecular interactions of neutral, closed-shell systems. This enables the simulation of a wide range of chemical systems, from molecular liquids and crystals to drug-like molecules and biopolymers.

The models were validated on a number of tasks, including the prediction of small-molecule torsion barriers, geometry optimizations, calculation of lattice parameters and enthalpies of the formation of molecular crystals, and calculation of Raman spectra of molecular crystals, including nuclear quantum effects. We also validated the models for predicting the densities and heats of vaporization of a range of molecular liquids. In particular, we study how well MACE-OFF reproduces the fundamental properties of water including density and radial distribution functions. To further showcase the capabilities of the model, we computed the free energy surface and J-coupling parameters of alanine tripeptide (Ala_3_) in vacuum and explicit water, simulated the folding of Ala_15_ at different temperatures, and carried out an all-atom simulation of the protein crambin in explicit water (18 000 atoms). Finally, we tested the computational speed of the current implementation in the LAMMPS and OpenMM simulation packages, demonstrating both strong and weak scaling.

While truly general-purpose force fields for biomolecular modeling require explicit treatment of long-range Coulomb interactions, the purpose of this paper is to push the capabilities of short-range potentials as far as they can go, in part as preparation for the inclusion of explicit, polarizable electrostatic interactions in the future.

## 2 Methods

### 2.1 MACE Architecture

The MACE model^2^ is a force field that maps the positions and chemical elements of atoms to the system’s potential energy. Linear scaling with system size is achieved by decomposing the total energy into atomic site energies. First, a graph is defined by connecting two nodes (atoms) by an edge if they are in each other’s local environment. The local environment N(i) is the set of all atoms *j* around the central atom *i* for which ∥**r**_*ij*_∥ ≤ *r*_cut_, where **r**_*ij*_ is the vector from atom *i* to atom *j*, and *r*_cut_ is the cutoff hyperparameter. The array of features of node *i* is denoted by **h**_*i*_^(*t*)^ and is expressed in the spherical harmonic basis, with its elements indexed by *l* and *m*. The superscript in *t* indicates the iteration steps (corresponding to “layers” of message passing in the language of graph neural networks). All MACE-OFF models are made up of two layers. The **h**_*i*_^(*t*)^ features depend on the chemical environment of the atoms for *t* > 0.

The node features **h**_*i*_^(0)^ are initialized as a (learnable) embedding of the chemical elements with atomic numbers *z*_*i*_ into *k* channels:

$$h_{i,k00}^{\left(0\right)}=\sum_{z}W_{kz}\delta_{zz_{i}}$$ 1

The superscript (0) in this case indicates the initial (0-th) layer. This type of mapping has been widely applied to graph neural networks^42–44^ and other models.^45,46^

Next, for each atom, the features of its neighbors are combined with the interatomic displacement vectors, **r**_*ij*_, to form the one-particle basis ϕ_*ij*,kη_1_ *l*_3_ *m*_3__^(*t*)^. The radial distance is used as an input into a learnable radial function *R*(**r**_*ij*_) with several outputs that correspond to the different ways in which the displacement vector and node features can be combined while preserving equivariance:^47^

$$\phi_{ij,k\eta_{1}l_{3}m_{3}}^{\left(t\right)}=\sum_{l_{1}l_{2}m_{1}m_{2}}C_{\eta_{1},l_{1}m_{1}l_{2}m_{2}}^{l_{3}m_{3}}R_{k\eta_{1}l_{1}l_{2}l_{3}}^{\left(t\right)}(r_{ij})Y_{l_{1}}^{m_{1}}({\hat{r}}_{ij})h_{j,kl_{2}m_{2}}^{\left(t\right)}$$ 2

where *Y*_*l*_^*m*^ are the spherical harmonics, and *C*_η_1_,*l*_1_ *m*_1_ *l*_2_ *m*_2__^*l*_3_ *m*_3_^ denote the Clebsch–Gordan coefficients. There are multiple ways of constructing an equivariant combination with a given symmetry (*l*_3_, *m*_3_), and these multiplicities are enumerated by the index η_1_.^48^

The one-particle basis ϕ is summed over the neighborhood, and the *k* channels are mixed together with learnable weights to form the permutation-invariant atomic basis *A*_*i*_:

$$A_{i,kl_{3}m_{3}}^{\left(t\right)}=\sum_{\tilde{k},\eta_{1}}W_{k\tilde{k}\eta_{1}l_{3}}^{\left(t\right)}\sum_{j\in N(i)}\phi_{ij,\tilde{k}\eta_{1}l_{3}m_{3}}^{\left(t\right)}$$ 3

Higher-order (many-body) symmetric features are created on each atom *i* by taking tensor products of the atomic basis, *A*_*i*_, with itself ν times, resulting in the “product basis”. The product basis is then contracted with generalized Clebsch–Gordan coefficients, $C_{\eta_{\nu }lm}^{LM}$ , to obtain the equivariant many-body basis, ***B***_*i*_:^48^

$$B_{i,\eta_{\nu }kLM}^{\left(t\right),\nu }=\underset{lm}{\sum }C_{\eta_{\nu }lm}^{LM}\overset{\nu }{\underset{\xi =1}{\prod }}A_{i,kl_{\xi }m_{\xi }}^{\left(t\right)}$$ 4

where bold ***l*** ***m*** denotes the ν-tuple of *l* and *m* values and, similarly to eq 2, η_ν_ enumerates the number of possible couplings to create the features with equivariance *LM*. The maximum body order is controlled by the parameter ν and is fixed at 3 (corresponding to 4-body terms, which include the central atom) for all MACE-OFF models in this work.

Finally, a “message” *m*_*i*_ is created on each atom as a learnable linear combination of the equivariant many-body features:

$$m_{i,kLM}^{\left(t\right)}=\sum_{\nu }\sum_{\eta_{\nu }}W_{z_{i}\eta_{\nu }kL}^{(t),\nu }B_{i,\eta_{\nu }kLM}^{(t),\nu }$$ 5

The recursive update of the node features (*t*: 0 → 2) is obtained by adding the message to the atoms’ features from the previous iteration, with weights that depend on the chemical element (*z*_*i*_):

$$h_{i,kLM}^{\left(t+1\right)}=\underset{\overset{\sim }{k}}{\sum }W_{kL,\mathrm{k̃}}^{\left(t\right)}m_{i,\mathrm{k̃}LM}^{\left(t\right)}+\underset{\overset{\sim }{k}}{\sum }W_{kz_{i}L,\mathrm{k̃}}^{\left(t\right)}h_{i,\mathrm{k̃}LM}^{\left(t\right)}$$ 6

Since the initial node features **h** ^(0)^ are dependent only on the chemical element of the atom, the second term of eq 6 is not included in the first layer. This makes it possible to set the energy of isolated atoms (that is, those with no neighbors) exactly,^49^ which is often desirable.^37,50^ The MACE models in this work have an effective receptive field of 2 × *r*_cut_ due to the two layers of message passing.

The site energy is a sum of read-out functions applied to the node features from the first and second layers. The read-out function is defined as a linear combination of rotationally invariant node features for the first layer and as a multilayer perceptron (MLP) for the second layer:

$$E_{i}=\sum_{t=1}^{2}E_{i}^{\left(t\right)}=\sum_{t=1}^{2}R^{\left(t\right)}(h_{i}^{\left(t\right)})$$ 7

$$R^{\left(t\right)}(h_{i}^{\left(t\right)})=\{\begin{array}{ll} \sum_{k}W_{k}^{\left(t\right)}h_{i,k00}^{\left(t\right)} & \mathrm{for}\;t=1\\ \mathrm{MLP}(\{h_{i,k00}^{\left(t\right)}{\}}_{k}) & \mathrm{for}\;t=2 \end{array}$$ 8

The forces and stresses on the atoms are calculated by taking analytical derivatives of the total potential energy with respect to the positions of the atoms and the components of the deformation tensor in the periodic setting, as is usual.

### 2.2 Training Data

The core of the training set is version 1 of the SPICE data set,^51^ with 95% of the data used for training and validation and 5% set aside for testing. Test/train splitting was performed at the molecule level, ensuring that conformers of the same molecule do not appear in both train/validation and test sets. Table 1 summarizes the training and test sets. The MACE-OFF models are trained to reproduce the energies and forces computed at the ωB97M-D3­(BJ)/def2-TZVPPD level of quantum mechanics,^52–56^ as implemented in the PSI4 software package.^57^ We have used a subset of SPICE that contains the 10 chemical elements, H, C, N, O, F, P, S, Cl, Br, and I, and has neutral formal charge (see Table 2 in ref 51 for element coverage in the data set). We have also removed the ion pair subset. Overall, we used about 85% of the full SPICE data set. The geometries in the SPICE data set have been generated by running molecular dynamics simulations using classical force fields,^58^ performed at both 300 and 500 K, and sampling maximally different conformations from the resulting trajectories.^51^

**Table 1:** Summary of Training and Test Sets^a^

	PubChem	DES370 K monomers	DES370 K dimers^59^	dipeptides	solvated amino acids	water	QMugs^60^	tripeptides
chemical elements	H, C, N, O, F, P, S, Cl, Br, I	H, C, N, O, F, P, S, Cl, Br, I	H, C, N, O, F, P, S, Cl, Br, I	H, C, N, O, S	H, C, N, O, S	H, O	H, C, N, O, F, P, S, Cl, Br, I	H, C, N, O
system size	3–50	3–22	4–34	26–60	79–96	3–150	51–90	30–69
# train	646821	16861	263065	19773	948	1597	2748	0
# test	33884	889	13896	1025	52	84	144	898

a The columns ‘PubChem’ to ‘Solvated Amino Acids’ correspond to the original SPICE dataset.^51^

The SPICE data set only contains small molecules of up to 50 atoms. To facilitate the learning of intramolecular nonbonded interactions, we augmented SPICE with larger 50–90 atom molecules randomly selected from the QMugs data set.^60^ The geometries were generated by running molecular dynamics simulations using GFN2-xTB,^23^ similarly to the protocol described in ref 46. The energies and forces were re-evaluated at the level of QM theory used in SPICE. Finally, to obtain a better description of water, the data set was further augmented with a number of water clusters carved out of molecular dynamics simulations of liquid water,^61^ with sizes of up to 50 water molecules. In addition to 5% of each subset, part of the COMP6 tripeptide data set^25^ was also recomputed at the SPICE level of theory and used as part of the test evaluation, but not for training.

In order to understand the role additional data plays in the performance of the model, we also investigated the difference in performance due to inclusion of additional configurations released as part of the second version of SPICE.^62^ The updated data set, which we incorporate into the MACE-OFF24­(M) model (see Section 2.3), contains, among others, additional 208,000 configurations comprising solvated PubChem molecules and amino acid-ligand pairs. The former were sampled from molecular dynamics using the same protocol as in the original data set, while the amino acid-ligand pairs were extracted from structures in the PDB. This release also included 1000 water clusters, containing up to 90 atoms. These were also added and used to complement the set of water clusters already included in the extended data set.

### 2.3 Model Details

The MACE model has parameters that enable the systematic control of model expressivity (and subsequent accuracy) against computational cost. In this paper, we present three variants of the MACE-OFF23 model, a small, a medium, and a large one, denoted in the text as MACE-OFF23­(S), MACE-OFF23­(M), and MACE-OFF23­(L), respectively. The models get more accurate with size but also have an increasing computational cost. The small model is well-suited for large-scale simulations, the medium model offers a good balance of speed and accuracy, and the large model is best used for small systems or when the highest possible accuracy is desirable. MACE-OFF24­(M) is an update to the medium model that uses an extended 6 Å cutoff and includes additional configurations from version 2 of the SPICE data set.^62^ The hyperparameters of the models are displayed in Table 2. All models were trained using the PyTorch^63^ implementation of MACE, available at https://github.com/ACEsuit/mace. More information about the training is provided in Section S1.

**Table 2:** Hyperparameters of the MACE-OFF Models

	23(S)	23(M)	23(L)	24(M)
cutoff radius (Å)	4.5	5.0	5.0	6.0
chemical channels *k* (see eq 1)	96	128	192	128
max L (see eq 5)	0	1	2	1
SPICE version	1	1	1	2

## 3 Results

### 3.1 Extended SPICE Test Set

First, we look at the pointwise errors of the energy and force predictions on a held-out test set for each of the three baseline MACE-OFF23 models. Figure 1 shows the per-atom energy, force, and intermolecular force root-mean-square errors (full statistics are provided in Section S2). As the size of the model increases, the models gradually become more accurate, with the large model generally achieving errors of around 0.5–1.0 meV/atom and 15–20 meV/Å, well below the 1 kcal/mol (43 meV) chemical accuracy limit for the drug-like organic molecules studied here.

**Figure 1:**
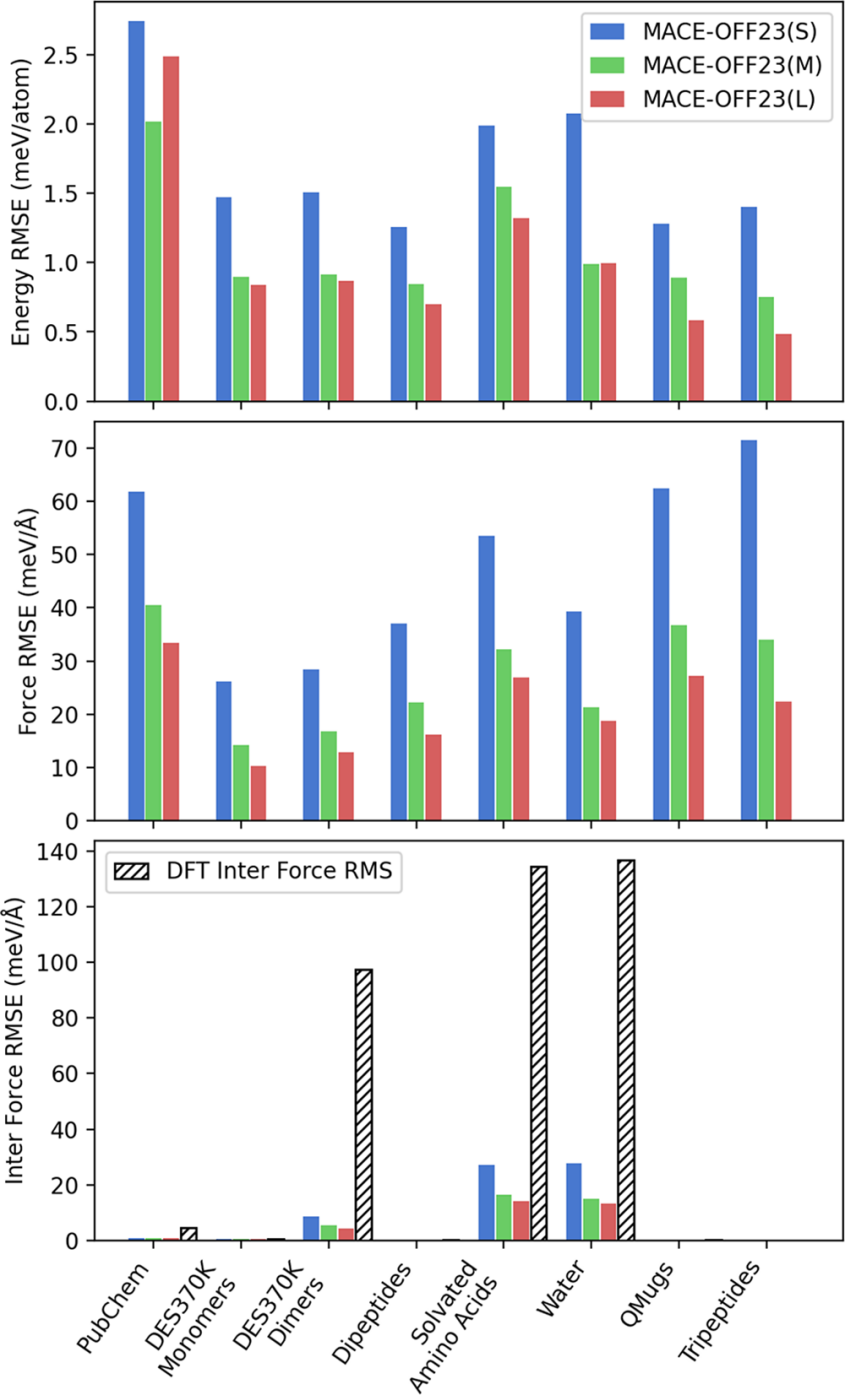
Test set root-mean-square errors (RMSE). Errors in the MACE-OFF23 models compared to the underlying DFT reference data, highlighting the relative accuracy of the three models. Bottom panels show specifically intermolecular force errors compared to overall DFT intermolecular force magnitudes (RMS). Note that for subsets comprising only single-molecule configurations (DES370 K Monomers, Dipeptides, QMugs, Tripeptides), intermolecular contributions are expected to be zero. The slight deviation from zero arises because DFT forces do not obey translational and rotational symmetries with sufficient accuracy, while MACE models do.

The last column represents an extrapolation task, the training set contains only dipeptides, and this test set looks at tripeptides, indicating that the models are able to extrapolate to larger fragments with more complex interactions, with no loss of accuracy.

The bottom panel of Figure 1 shows specifically the intermolecular force errors, which were obtained by separating the force contributions to molecular translations and rotations (see Section S2 and ref 64 for details). These interactions are crucial, as they underpin the thermodynamics and transport properties in the organic condensed phase. MACE-OFF23­(M) and MACE-OFF23­(L) both yield similar errors of around 5–15 meV/Å, which are about 1.5–3 times smaller than total force errors, and about 5–10% of the typical DFT intermolecular force magnitude (RMS). For context, relative intramolecular force errors, which are roughly equivalent to total force errors, are around 1–2%. The latter are routinely used across the literature to benchmark ML models; however, we advocate that intermolecular RMSE errors are a more appropriate metric to assess model accuracy for organic condensed-phase applications. The results here demonstrate the remarkable accuracy of the MACE models in predicting these subtle but crucial interactions. We will investigate how these energy and force errors translate into condensed-phase physical property predictions in later sections.

### 3.2 Dihedral Scans

Next, we evaluate the performance of the MACE-OFF23 models on dihedral scans of drug-like molecules. This task is routinely carried out using quantum mechanical methods to create reference data for parametrizing classical empirical force field dihedral terms.^65^ The task is particularly challenging, as constrained geometry optimizations can be difficult to converge if the potential energy surface is not smooth, as has been observed, for example, for the ANI-1x potential.^66^

#### 3.2.1 TorsionNet-500

The top panel of Figure 2 summarizes the results for the TorsionNet-500 data set.^68^ This data set contains torsion drives of 500 different molecules, selected to cover a wide range of pharmaceutically relevant chemical spaces. The original data set was reported at the B3LYP/6–31G­(d) level of DFT theory. For consistency with the SPICE data set, we recreated the torsion profiles using the DFT setting of SPICE, which uses a higher level of theory and basis set (Section S3).

**Figure 2:**
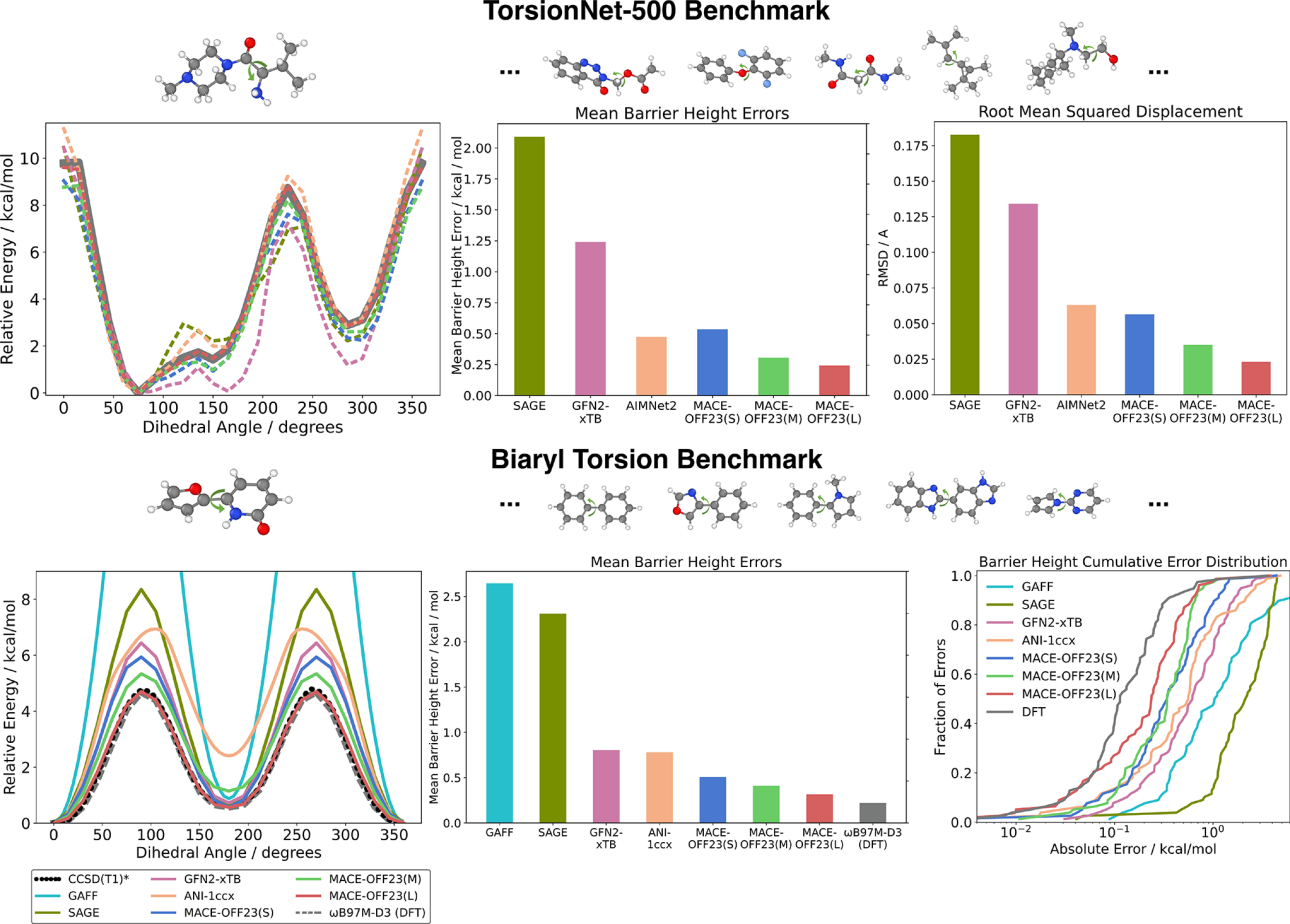
Dihedral benchmark scans. The top panel shows torsion drive data for the TorsionNet-500 data set, which has a wide chemical diversity (five example molecules are shown). The bottom panel focuses on the torsion angle between two aromatic rings in the biaryl torsion benchmark,^67^ which contains 78 molecules (five examples are shown).

The first panel shows an example of a torsion drive, indicating the complex energy profile that the MACE models are able to capture closely, including geometries far from equilibrium. The center panel shows the mean barrier height error of a number of representative models, comparing the Sage classical empirical force field,^21^ a semiempirical quantum mechanical method GFN2-xTB,^23^ a recent transferable machine learning force field AIMNet2,^8^ and the three MACE-OFF23 models. Again, systematic improvements in accuracy with MACE model size are observed, with medium and large models, in particular, achieving errors of around 0.25 kcal/mol, compared to the reference method. The AIMNet2 model achieves accuracy comparable to that of the small MACE-OFF23 model. A similar conclusion can be drawn from the comparison of the molecular geometries by looking at the root mean squared deviation of the atomic positions averaged over the full torsion scans, as indicated by the top right panel of Figure 2. This shows that MACE-optimized geometries have a deviation of about 0.025 Å, meaning they are almost indistinguishable from DFT-optimized structures. It is important to note that the other models were trained to different levels of DFT, which might also contribute to the observed differences.^69^

#### 3.2.2 Biaryl Fragments

The biaryl torsion benchmark investigates the torsional profiles of about 100 different biaryl fragments computed at the coupled cluster level of theory.^67^ In these molecules, the rotatable bond is between two aromatic rings. Such chemistry frequently occurs in drug-like molecules, and the profiles are typically challenging to model accurately using empirical classical force fields. Following previous studies, we used a subset of 78 molecules to facilitate comparisons with the ANI-1ccx model, which is only parametrized for H, C, N, and O chemical elements.^67^

In the bottom panel of Figure 2, we compare the results of torsion drives of empirical force fields, semiempirical QM methods, and the ANI-1ccx machine learning force field^26^ with the MACE-OFF23 models. We also compare the DFT potential energy surfaces (using the SPICE level of theory) with the published gold standard coupled cluster data.

The DFT torsion drives achieve a mean barrier height error of 0.2 kcal/mol with respect to coupled cluster data and are therefore the best result theoretically possible for MACE-OFF23 models, which were trained on the same DFT level of theory. We find that the large model comes close to this, with a mean absolute error of 0.3 kcal/mol. The medium and small MACE models have barrier height errors of 0.4 and 0.5 kcal/mol, respectively. Remarkably, the small MACE model is significantly more accurate than the next-best non-DFT methods, ANI-1ccx, and GFN2-xTB, as illustrated in the bottom-center plot of Figure 2. In particular, unlike MACE-OFF23, coupled cluster reference calculations were used to parametrize the ANI-1ccx and GFN2 models. In the bottom right, we also show the cumulative error distributions to verify that the MACE-OFF23 barrier height errors are not only accurate on average but also robust, with essentially no outliers.

#### 3.2.3 Infrared Spectroscopy of a Drug Molecule

To assess the accuracy of the MACE-OFF23­(M) model for finite temperature properties of molecules, we studied the infrared spectrum of an isolated paracetamol molecule at room temperature. Molecular dipole moments are predicted using the MACE architecture, trained on the SPICE data set, and quantum nuclear effects are incorporated using path-integral coarse-grained simulations (PIGS).^70^ Full details are provided in Section S8. This is a stringent test as reproducing the line positions and their relative intensities requires an accurate treatment of both intramolecular interactions and the dipole moment in out-of-equilibrium configurations. As shown in Figure 3, we estimate the classical and approximate quantum IR spectra^70^ and compare them directly with experimental data at 293 K.^71^ For low and intermediate frequencies (up to around 2000 cm^–1^), the classical and quantum spectra agree with each other and with the experimental data, allowing the identification of all collective modes. For the high-frequency O–H and N–H modes, we observe that the classical spectra are blue-shifted due to the absence of anharmonic zero-point fluctuations, which typically soften these bonds. A quantum treatment of nuclear dynamics results in good agreement with the experimental data, with the exception of a net blue shift and a slight discrepancy in the relative intensities of the high-frequency stretching with respect to the lower-frequency modes, likely due to the limitations of the reference density functional.

**Figure 3:**
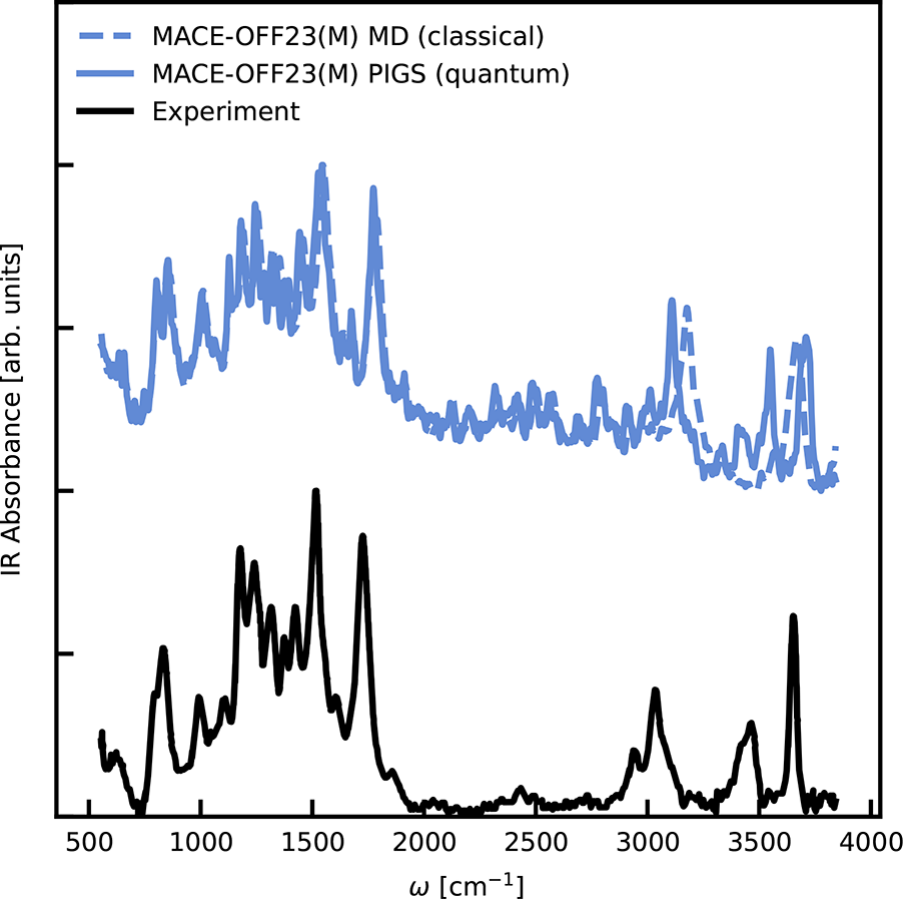
Infrared (IR) spectrum of a paracetamol molecule. The classical (blue dashed) and quantum (blue solid) IR spectra at 293 K using the MACE-OFF23­(M) model are computed by propagating the system and estimating the time correlation function of the time derivative of the total dipole moment. The experimental data are taken from ref 71.

### 3.3 Molecular Crystals and Organic Liquids

In the following, we demonstrate the ability of the MACE-OFF force fields to simulate the vibrational and thermal properties of molecular crystals. Based on relative accuracy and computational expense, in what follows, we present data only for the medium MACE-OFF models. Additional experiments, where either the higher throughput of the small model or the additional accuracy of the large model is desired, are reported in the Supporting Information.

#### 3.3.1 Lattice Enthalphies

We assessed the ability of the MACE-OFF23­(M) model to describe the stability of molecular crystals. We computed the enthalpies of the sublimation of a range of 23 representative small molecular crystals^72^ following the protocol of ref 73 (Section S4).

Figure 4 compares the predicted sublimation enthalpies to the experimentally measured ones. This task is often used to test various DFT functionals,^72^ as well as beyond-DFT methods^74^ and tuned machine learning potentials.^75^ Since the ωB97M-D3­(BJ) functional used to parametrize MACE-OFF23 does not have periodic implementation, an estimate of the highest achievable accuracy is not available. The figure shows that the MACE-OFF23­(M) model is able to capture trends and improve significantly compared to the ANI-2x model. The mean absolute error of 1.8 kcal/mol is comparable to the errors of several different dispersion-corrected density functionals for a fraction of the computational cost.^72^ The sublimation enthalpies for all MACE-OFF models are tabulated in Section S4, and we observe that the small model is intermediate in accuracy between the medium model and ANI-2x, while the large model does not give any significant performance improvement.

**Figure 4:**
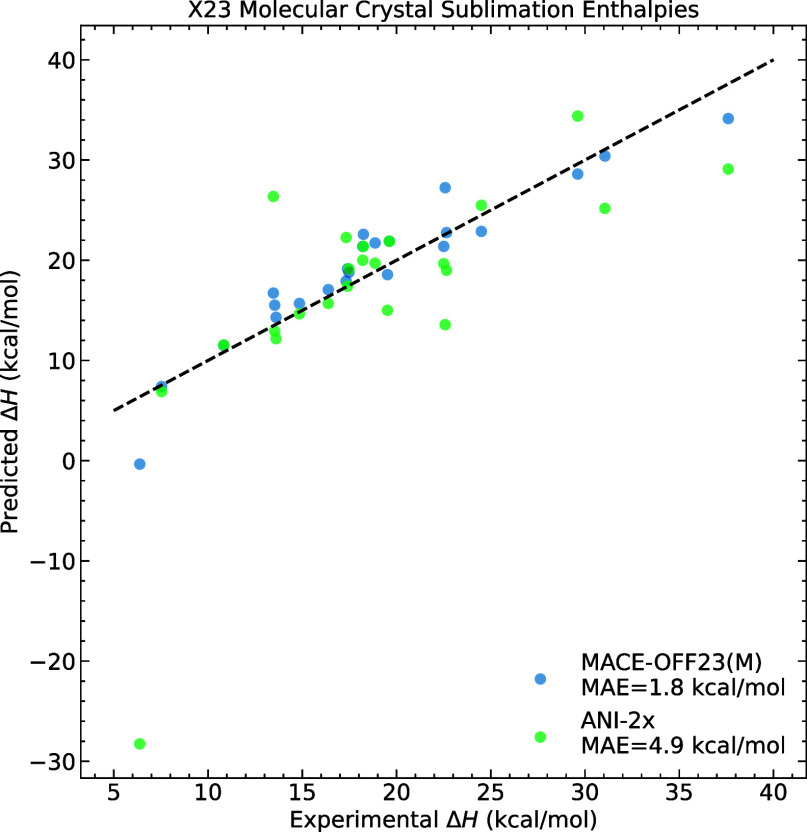
Sublimation enthalpies of molecular crystals. Comparison between predicted sublimation enthalpies of the MACE-OFF23­(M) and ANI models and experiment.

Additional analyses of the relaxed unit cell vectors using the MACE-OFF23­(L) model and the powder Raman spectrum of crystalline paracetamol are presented in Sections S5 and S8.

#### 3.3.2 Condensed-Phase Properties of Organic Liquids

Small-molecule empirical force fields are fitted to reproduce experimental condensed-phase properties, including densities and heats of vaporization. As such, errors on small molecular systems are expected to be well within chemical accuracy.^21,76^ For machine learning force fields, this task represents a greater challenge since the potential is typically trained only using QM reference data for isolated molecules and molecular dimers. From these ab initio data, the models must learn the long-range interactions necessary to reproduce bulk properties without fitting to them.

First, we investigate the predictions of molecular liquid densities under ambient conditions using the medium MACE model shown in the top panel of Figure 5. MACE-OFF23­(M) achieves an MAE of 0.09 g/cm^3^, indicating that it can generalize from the intermolecular interactions between molecular dimers in the training set to the condensed phase, retaining a good correlation with the experiment. In contrast, the ANI-2x potential has significantly higher errors, with MAE and RMSE errors twice as large as those of MACE-OFF23­(M) (Section S6).

**Figure 5:**
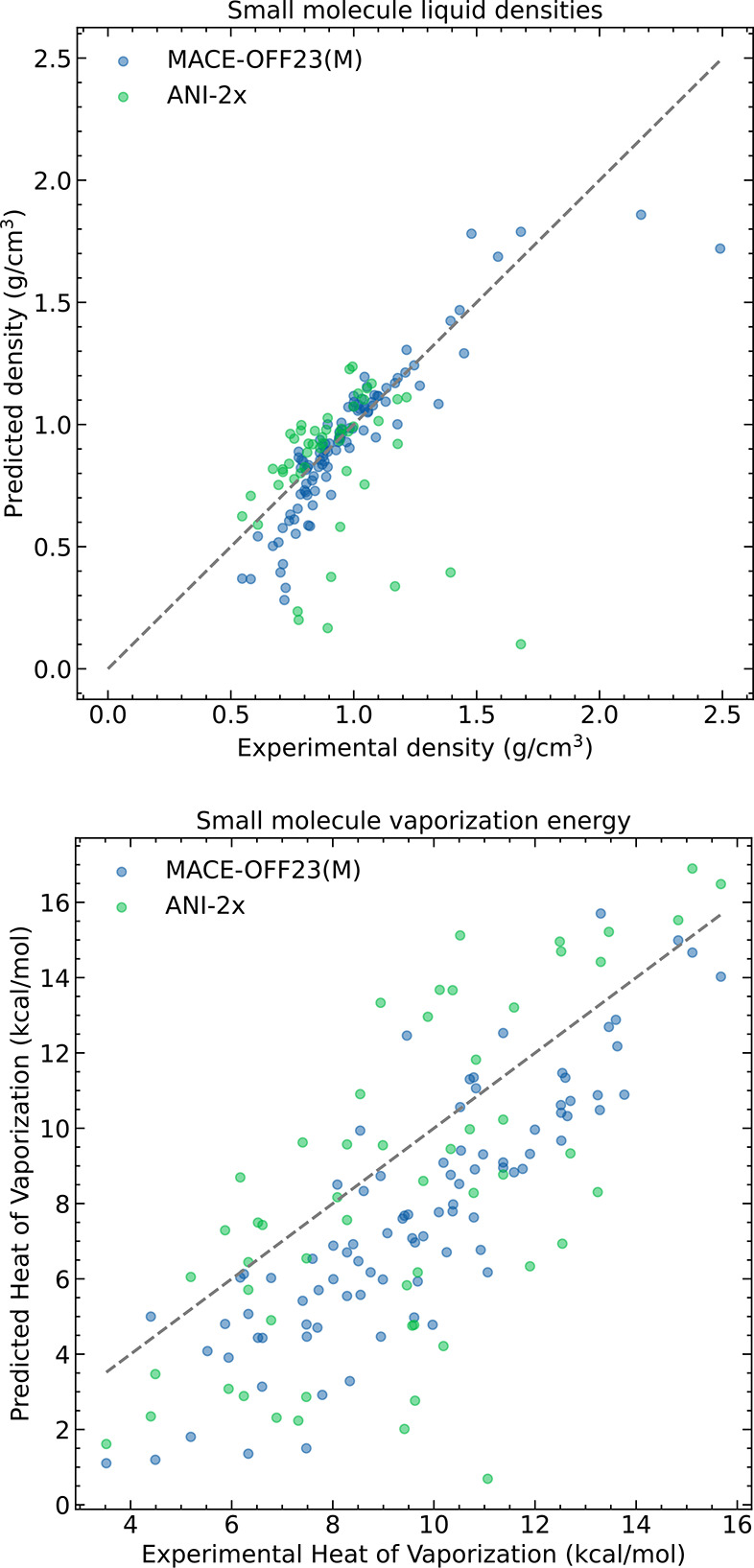
Molecular liquids. Comparison between MACE-OFF23­(M) and ANI-2x with experiment for densities (top) and heats of vaporization (bottom) of condensed-phase organic liquids.

To investigate the performance of the MACE-OFF23 models in the condensed phase, we selected a benchmark set of 109 molecules from ref 77, containing a representative set of functional groups relevant to medicinal chemistry and biology. For the liquid boxes containing approximately 600 atoms, MACE-OFF23­(S) achieved a throughput of 2.1 × 10^6^ steps/day via OpenMM on a single 80GB NVIDIA A100 GPU, while the medium model achieved a throughput of 1.1 × 10^6^ steps/day. Meanwhile, MACE-OFF23­(L) achieved a throughput of 2.8 × 10^5^ steps/day on the same system, highlighting the significant overhead compared with the other two models. See Section 3.5 for a further discussion of the computational performance.

The vast majority of the outliers in the density predictions can be assigned as either low-boiling-point ether-containing compounds, polychlorinated hydrocarbons, or dibromo-containing compounds. In the first case, this may be due to a marginal underprediction of the boiling point, since these compounds were simulated at 10 K below their tabulated boiling points. In the latter two cases, this functional group dependence suggests that these nonbonded interactions are insufficiently represented in the present training set, which contains, for example, only 84 examples of dibromo-containing dimers. This highlights the need for additional coverage of specific functional groups within SPICE (for example, the recent AIMNet2 data set includes 20 million configurations to cover 14 chemical elements^8^).

We further investigated the performance of MACE-OFF23­(M) on the heat of vaporization. The predictions correlate strongly with the experimental data; however, there is a systematic offset of approximately 2 kcal/mol (Figure 5). We hypothesized that this may result from an underprediction of the total intermolecular interactions originating from two sources: those that appear in molecular dynamics simulations outside the effective cutoff of the MACE model (compare classical simulations, which typically employ long-range corrections to the total energy^78^) and those that the model has not learned from the limited set of intermolecular interactions in the training set.

The effect of the choice of the MACE-OFF model on the condensed-phase properties is further investigated in Section S6. In general, we observe that the small model systematically overpredicts the experimental density (MAE 0.23 g/cm^3^), which is consistent with its larger intermolecular force errors (Table S1). We did not observe any significant improvements in accuracy, over the medium model, using either the large model or a model trained with an extended cutoff of 6 Å. This further reinforces the hypothesis that the poorly predicted densities are a result of under-representation of the functional groups in the training set. Nevertheless, these data indicate that, given a reasonable receptive field and a sufficiently expressive model, it is possible to fit a local force field that captures the interactions required to recover experimental properties. Further improvements in accuracy are possible without resorting to fitting to experiment by further increasing the size of the training data set and the explicit addition of long-range Coulomb interactions into the model, which we leave for future work.

### 3.4 Biomolecular Simulations

In the following section, we benchmark the performance of the MACE-OFF models on several well-studied large-scale biomolecular systems. This particular application area is of key interest, and state-of-the-art classical protein force fields have been carefully parametrized over many decades to reproduce key quantum mechanical and thermodynamic properties. However, this extensive parametrization process makes it difficult to extend classical force fields, and overall accuracy and transferability remain fundamentally limited by their functional form. This typically becomes apparent only when each generation of advancing hardware enables sufficiently long simulations to identify the issues.

#### 3.4.1 Water Structure and Dynamics

A key requirement for bio-organic force fields is an accurate description of water. Several empirical water models have been developed for this purpose, with the most common for all-atom biomolecular simulations being TIP3P. Figure 6 shows that MACE-OFF23­(M) correctly predicts the radial distribution function (RDF) of water, performing comparably with both TIP3P and the highly accurate MB-pol water model, whose RDF is indistinguishable from the experiment.^79^ These interactions are modeled in a purely local way by MACE, having been trained on small water clusters containing up to 50 water molecules. This result further emphasizes the ability of the model to generalize to bulk simulations, even though the training data only contained nonperiodic clusters. In contrast, ANI-2X shows a significant overstructuring of the RDF; however, this is likely driven primarily by the lack of dispersion correction in the underlying quantum mechanical method used to label the training set. Additional tests of the vibrational density of states of liquid water are presented in Section S8 and show good agreement with the experiment.

**Figure 6:**
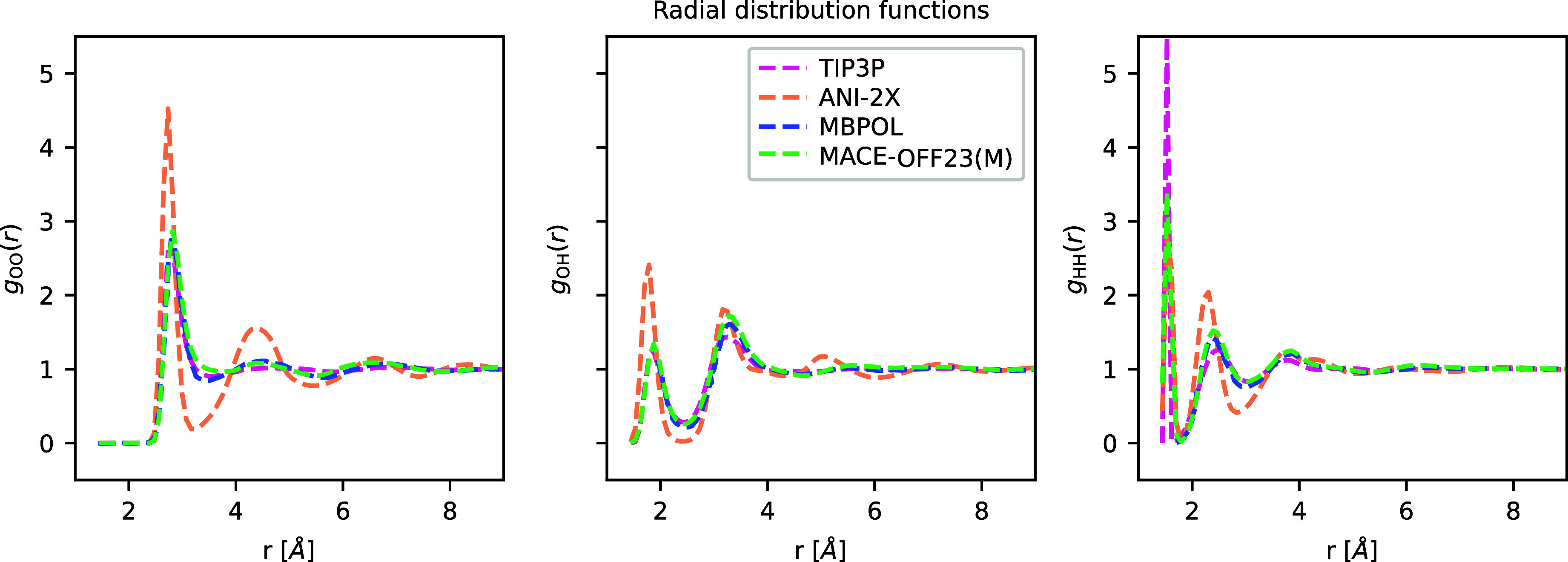
Water structure. Radial distribution functions of MACE, ANI, and TIP3P models. MB-pol traces are reproduced from ref 79.

We further calculated the temperature dependence of the water density using NPT molecular dynamics simulations, as shown in Figure 7. This is a highly sensitive test of the accuracy of intermolecular interactions.^64^ The MACE-OFF23­(M) model overestimates the density of liquid water by approximately 20%. Since there is extensive coverage of water clusters in the training set, we hypothesized that this results from a missing long-range contribution beyond the receptive field. To investigate this hypothesis, we fitted an additional model, which has an increased 6 Å layerwise cutoff compared with 5 Å. The larger receptive field leads to a predicted water density within 2% of the experimental value at room temperature and within 5% across the full temperature range (Figure 7). We note that similar errors are reported using many dispersion-corrected DFT exchange-correlation functionals.^80^ At this point in the model development, the second version of SPICE had been released, which included Solvated PubChem and Amino Acid-Ligand Pairs data sets.^62^ Given the direct applicability of these training data and the importance of modeling water to biomolecular simulations, the remainder of this section uses the MACE-OFF24­(M) model, which includes the additional training data and extended 6 Å cutoff (Table 2). To confirm no loss of performance on the incorporation of the additional training data, we confirmed that the test set errors are in agreement with MACE-OFF23­(M) (Section S2.1).

**Figure 7:**
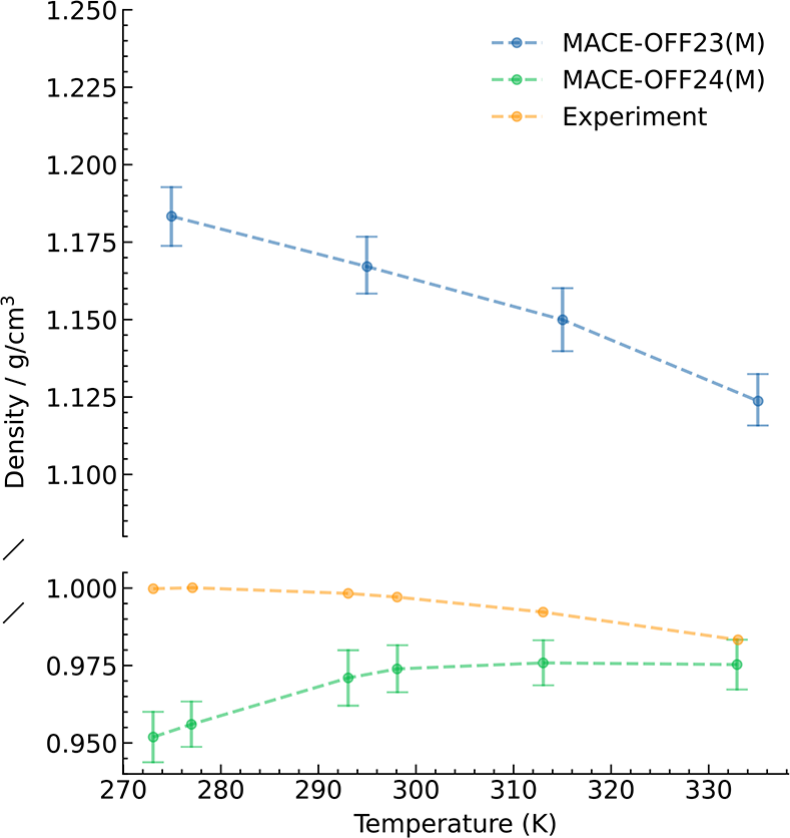
Water density as a function of temperature. Comparison between MACE-OFF23­(M) and the extended cutoff MACE-OFF24­(M) model.

#### 3.4.2 Ala_3_ Free Energy Surface

We first investigated the ability of MACE-OFF24­(M) to reproduce the free energy surface of an Ala_3_ tripeptide. We observed a computational performance of 9.6 × 10^6^ steps/day for the vacuum system, and 2.2 × 10^6^ steps/day for the solvated system on an NVIDIA 80GB A100 GPU via the OpenMM interface. Although this remains significantly more expensive than the MM simulation (around 150 × 10^6^ steps/day), it nevertheless enables sufficient sampling of the free energy surface.

Figure 8 shows the free energy surface of Ala_3_ in water, modeled by AMBER14SB/TIP3P and MACE-OFF24­(M). Well-tempered metadynamics was used to enhance sampling along the central ϕ and ψ backbone angles (Section S7.1). The AMBER14SB simulation identifies four local minima, corresponding to the antiparallel β-sheet (ϕ < −120°, ψ > 120°), a right-handed α-helix (ϕ = −60°, ψ > −60°), the corresponding left-handed α-helix (ϕ = 60°, ψ > 60°), and a polyproline II (PPII)-type structure (ϕ = −60°, ψ > 120°). Comparing the dihedral angle distribution with NMR J-coupling constants, the AMBER14SB simulation is generally in good agreement, with a slight overpopulation of both α-helical structures and underpopulation of the PPII mesostate.^81^ We therefore use this to enable visual comparison with a well-tuned classical force field.

**Figure 8:**
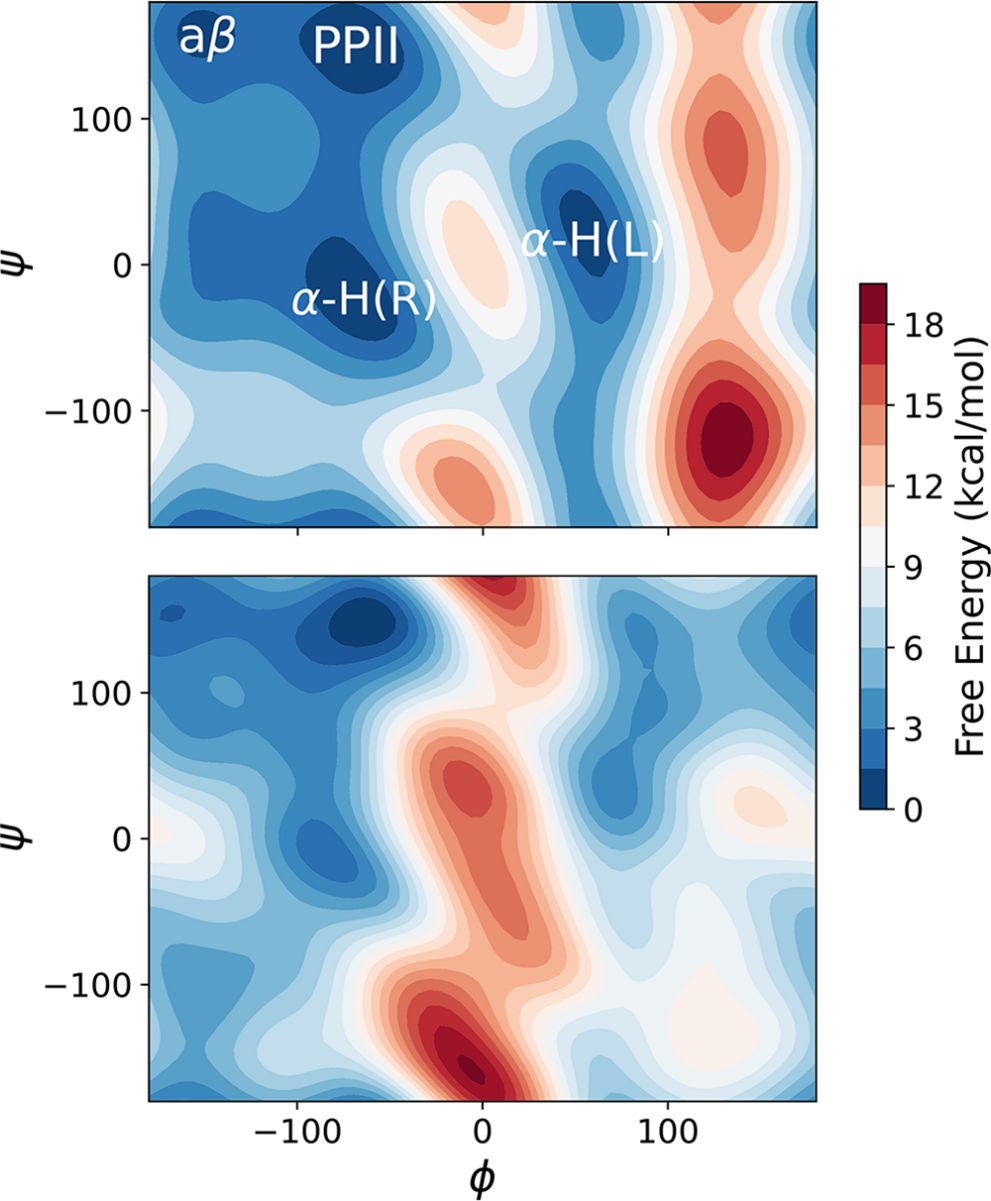
Free energy surfaces of Ala_3_ in explicit solvent. AMBER14SB/TIP3P (top) and MACE-OFF24­(M) (bottom).

The corresponding MACE simulations show a similar free energy surface to that of AMBER14SB/TIP3P, with the same four local minima identified. While the relative depths of the α-helical conformations are in agreement, AMBER predicts the free energy of the antiparallel β-sheet to be 1 kcal/mol higher than the PPII minimum, compared to 0.1 kcal/mol for MACE.

We also compare the unbiased conformational ensemble from 20 ns of NPT dynamics sampled with MACE-OFF24­(M) directly to the experiment by calculating ^3^J-coupling constants from the Ramachandran distribution. The computed values are shown in Table 3, in addition to literature values for AMBER14SB, ANI-2x, and experiment.^81–83^ MACE-OFF24­(M) accurately predicts coupling constants and is comparable with AMBER14SB. In agreement with our previous findings, both methods significantly outperform ANI-2x, highlighting the difficulties in computing accurate dynamics in the solution phase with MLPs.

**Table 3:** Summary of ^3^J Couplings Computed from Dihedral Distributions^a^

	^3^*J*(H_N_,H_α_)	^3^*J*(H_N_,C_β_)	^3^*J*(H_N_,C*′*)
MACE-OFF24(M)	5.70(0.43)	1.64(0.18)	1.37(0.30)
ANI-2x	7.5	1.2	1.8
AMBER14SB	6.07	1.87	1.13
EXP	5.68(0.11)	2.39(0.09)	1.13(0.08)

a AMBER14SB and experimental values are taken from ref 81, and ANI-2x data from ref 82. Standard errors in the mean from block averaging are shown in parentheses for MACE-OFF, and experimental uncertainties are reproduced from ref 81.

#### 3.4.3 Folding Dynamics of Ala_15_

Having confirmed that MACE-OFF24­(M) is capable of recovering a good approximation to the free energy surface of a small peptide, we next investigated the folding of the longer helical peptide Ala_15_. Because this is a system size significantly larger than the dipeptide configurations on which the model was trained, this represents a nontrivial test of the extrapolation capability of the potential, including its ability to capture complex hydrogen bonding interactions required to stabilize the secondary structure. Such simulations are believed to be particularly difficult with purely local models, such as those in the MACE-OFF family.

As an initial test, the fully extended Ala_15_ structure was simulated in vacuo. Figure 9 shows the assignment of the secondary structure during the simulation. The peptide folded within 200 ps, adopting initially a “wavy” intermediate structure before first folding into a 3_10_ helix. The secondary structure oscillates between the α-helix and the 3_10_ helix for the remainder of the simulation, with the α-helix being the predominant motif. These results are in agreement with other works,^9,39^ in which polyalanine peptides are observed to fold through a “wavy intermediate” and oscillate between the 3_10_ and α-helices. MACE-OFF24­(M) predicts the 3_10_ helix in a lower ratio than SO3LR and GEMS. The oscillatory behavior has also been observed experimentally in alanine-rich polypeptides.^84^

**Figure 9:**
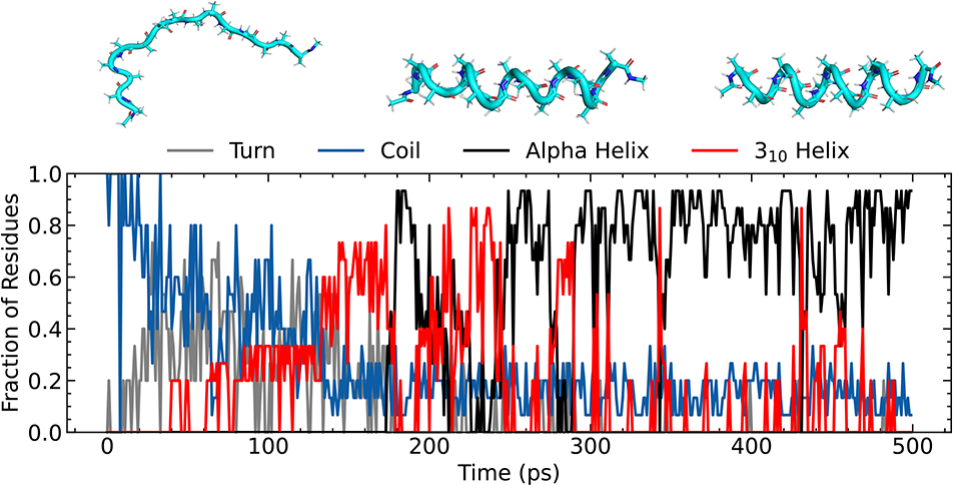
Ala_15_ folding dynamics. Secondary structure assignment during a 500 ps trajectory generated with MACE-OFF24­(M). The structure proceeds via a “wavy” intermediate and oscillates between the α- and 3_10_ helices. Secondary structure assignment was performed with the STRIDE algorithm.^85^

We also measured the secondary structure of the peptide as the temperature was increased. Starting from the folded structure at 300 K, the temperature was increased in steps from 300 to 900 K over the course of 3 ns (Figure 10). The folded structure remains stable up to around 500 K, after which point, the helix unfolds, and the structure is primarily a random coil.

**Figure 10:**
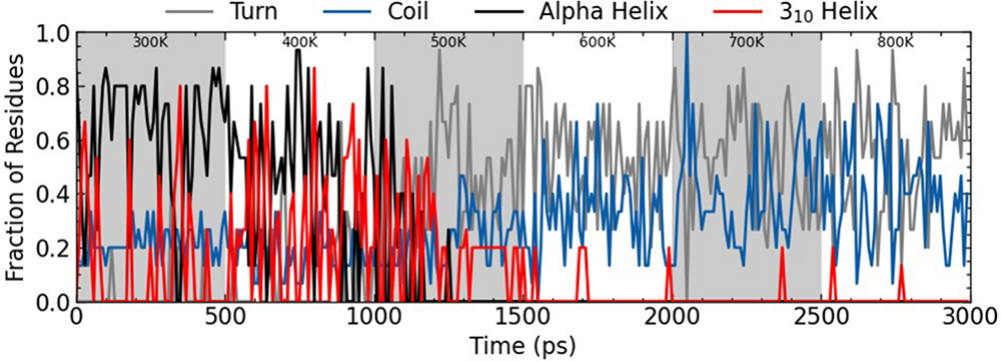
Ala_15_ temperature dependence. Secondary structure assignment with increasing temperature.

#### 3.4.4 Protein Simulation in Explicit Solvent

Finally, we investigated the simulation of a fully solvated protein with MACE-OFF. As a test case, we chose Crambin, a 42-residue threonine storage protein that contains four charged residues. This test goes significantly beyond what can typically be expected from a local force field trained on neutral species only.

Using the MACE-OFF24­(M) model, we first confirmed that the force field was indeed capable of simulating a large biomolecular system containing charged residues. In particular, it has recently been shown that the FENNIX potential fails to simulate a solvated 28 residue peptide due to unphysical proton transfers involving charged residues.^37^ We confirmed that the root-mean-square fluctuations of the protein backbone relative to the minimized structure remained below 1 Å for the duration of a 1 ns simulation, no bond breaking occurred, and the secondary structure motifs remained intact (Figure S2). We obtained a throughput of 3 × 10^5^ steps per day on a single NVIDIA A100 80GB GPU.

We then investigated the ability of MACE-OFF24­(M) to capture the key vibrational modes of the system. To this end, we prepared a simulation box containing crambin in the high hydration state, that is, fully solvated in explicit water, for a total system size of approximately 18,000 atoms. The power spectrum was calculated from the final 125 ps of a 1.6 ns simulation recorded at 2 fs resolution, to match the simulation length and sampling frequency employed in ref 41. This system has also been investigated with the AMBER classical force field and several machine learned potentials, including GEMS and, more recently, SO3LR.^9,39,40^ All three MLPs produce spectra that are in qualitative agreement with each other. The water peaks at 1640 and 3200–3600 cm^–1^ are reproduced reasonably accurately, especially considering that no nuclear quantum effects are taken into account (Figure 11). Furthermore, in the low-frequency region, two peaks are identified in the power spectrum by GEMS, SO3LR, and MACE-OFF24­(M). The most obvious of these occurs at 60 cm^–1^ and corresponds to localized internal side-chain fluctuations, while a peak at 220 cm^–1^ is due to specific configurations of water at the protein surface.

**Figure 11:**
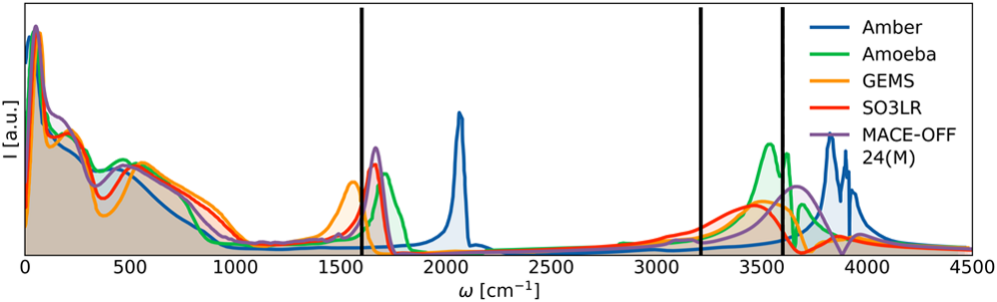
Protein in explicit solvent. Power spectrum of the high hydration state of crambin computed from the final 125 ps of a 1.6 ns simulation with MACE-OFF24­(M). GEMS, AMBER, and SO3LR spectra are reproduced from ref 41. Black lines denote experimental peaks at 1640 cm^–1^ and the range of 3200–3600 cm^–1^.

We note that, while a 125 ps trajectory is sufficient to qualitatively identify the spectral features, a much longer simulation of at least several nanoseconds would be required to fully converge the THz region of the spectrum and enable full quantitative comparison with experiment, as demonstrated for the GEMS model in ref 39. We note that the power spectrum computed on the final 1.3 ns simulation (discarding the initial 300 ps to equilibration) does not yield a significantly different spectrum to that of the 125 ps simulation used in Figure 11, indicating that significantly more sampling would be required to resolve the THz lineshapes sufficiently to enable experimental comparison.^86^

It is also noteworthy that while the three MLPs compared in this section give a qualitatively similar result, their modeling choices are significantly different. Most notably, GEMS and SO3LR explicitly include long-range interactions, while MACE is local. Additionally, of these models, both MACE and SO3LR are transferrable potentials, while GEMS is fitted in a system-specific fashion. We mention that, in addition to the GEMS model, the authors benchmarked a variant, which was trained only on small fragments, denoted GEMS*, instead of the large system-specific configurations used to fit GEMS.^39^ The power spectrum produced by this model is also in qualitative agreement with the other ML potentials; however, the RMSD is reduced, indicating that the protein structure is confined to small fluctuations around local minima. This model also fails to reproduce experimental observables of other biomolecular systems, for example, Ala_15_ is not predicted to fold under the GEMS* potential, suggesting that the top-down fragments are required to accurately reproduce nonbonded interactions within the GEMS family of models.

The results of Sections 3.3 and 3.4 highlight the most significant differences between previous transferable organic ML potentials and MACE-OFF models. While there is still significant work to be done to comprehensively show improvement over classical force fields for large biomolecular systems, these results demonstrate the capabilities of models fit ‘bottom up’ from quantum mechanical data to learn subtle intermolecular interactions and correctly predict macroscopic properties.

### 3.5 Computational Performance

To evaluate the computational performance of the MACE-OFF models, we performed NVT molecular dynamics for water using both OpenMM and LAMMPS, with simulation boxes ranging from 1 × 10^2^ to 1 × 10^5^ atoms. The OpenMM results shown in Figure 12 were collected with a single NVIDIA A100 80GB GPU. The LAMMPS results shown in Section S9 highlight both CPU and GPU evaluation.

**Figure 12:**
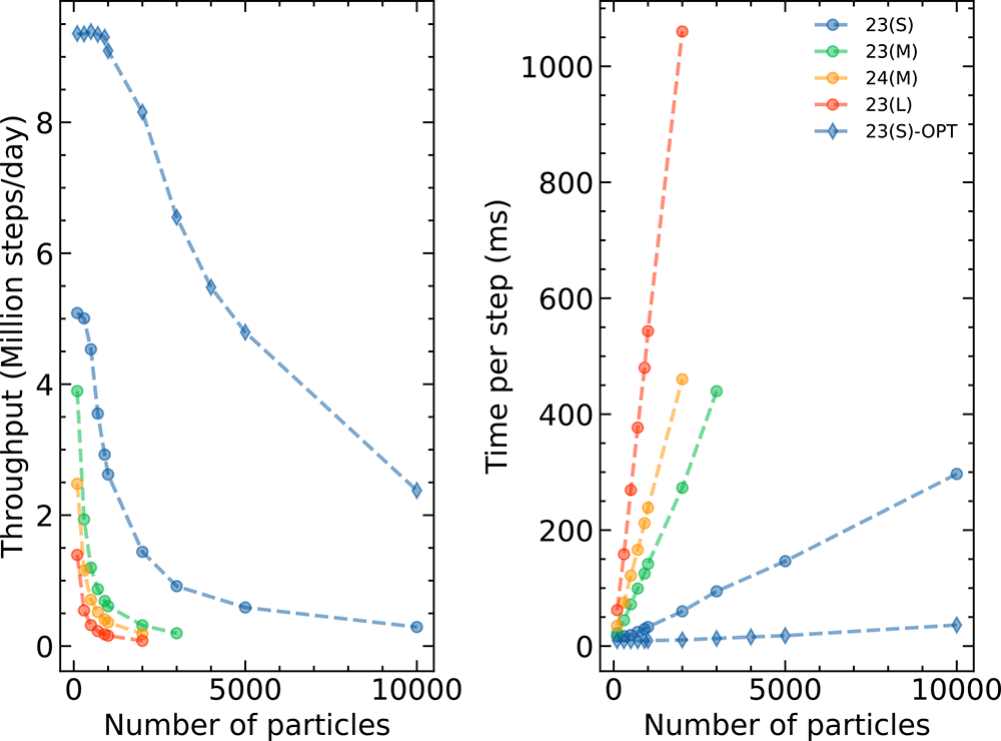
Performance in OpenMM. Performance of MACE-OFF models in OpenMM for liquid water in the NVT ensemble at 1 g/cm^3^ at 300 K with a 1 fs time step on a single NVIDIA 80GB A100 GPU. The cuda_mace implementation is labeled ‘OPT’. Forces were evaluated in double precision and integrated in single precision.

In addition to the native PyTorch implementations of the MACE-OFF23 models, we also report results for optimized implementations leveraging custom C++, CUDA, and Kokkos code. One optimized variant incorporates custom CUDA kernels, which are packaged as a standalone, inference-only library, cuda_mace.^87^ Another pure-C++ implementation, as well as a Kokkos implementation, will be made available as part of the Symmetrix library.^88^

In these optimized variants, the learnable radial function *R*_*k*η_1_ *l*_1_ *l*_2_ *l*_3__^(*t*)^(**r**_*ij*_) is evaluated with cubic splines, which efficiently map **r**_*ij*_ to each of the outputs of the radial MLP. Furthermore, eqs 2 and 3 are implemented in unified kernels that simultaneously compute the one-particle basis and perform the summation over neighbors to generate the invariant atomic basis *A*_*i*_. The computation of spherical harmonics, as detailed in eq 2, is accelerated using the sphericart^89^ library, which offers a highly optimized implementation for real spherical harmonics. All linear and equivariant linear operations have been substituted with custom kernels, and the cuda_mace implementation further leverages tensor core acceleration, while incorporating error-correction techniques to ensure numerical accuracy. Additionally, the computation of the product basis **B**_*i*_ in eq 4 is optimized using the sparsity of the generalized Clebsch–Gordon coefficients $C_{\eta_{\nu }lm}^{LM}$ , significantly accelerating the product calculations over both the body order *v* and the coupling terms.

## 4 Outlook

In this paper, we presented a series of MACE-OFF force field models, demonstrating the broad applicability, transferability, and capability of purely short-range machine learning potentials for organic (bio)­molecular simulations. We have shown that the models, based on the MACE higher-order equivariant message-passing architecture, can improve on the pioneering ANI models^27^ that served as the only widely applicable machine learning force fields for molecules for a number of years. While a full ablation study between different ML models is not possible due to a wide range of design choices made in their construction, the MACE-OFF model architecture, training data, and loss function all contribute to significant improvements in both accuracy and extrapolation, combined with high computational speed.

We trained here so-called small, medium, and large versions of the MACE-OFF models, with systematically improving accuracy when benchmarked against gas-phase quantum mechanical data. We further investigated the accuracy of the medium models against a range of crystalline, liquid, and biomolecular condensed-phase data, as we found them to provide a good compromise between accuracy and computational expense. We have made all force fields available to the community so that users can choose the model most suited to their application.

The lack of explicit long-range interactions limits the domain of applicability of the present model to neutral, nonradical, and nonreactive systems. This is something that the recently published AIMNet2 model aims to address by extending the ANI models to include charged species and long-range interactions.^8^ We are currently working on a next-generation MACE-OFF model that will similarly include an explicit description of charges, enabling the description of amino acids with different protonation states, charged nucleic acids, and counterions. This will pave the way toward obtaining an accurate quantum mechanical transferable machine learning force field for simulating the full range of biologically relevant systems.

## Supplementary Material



## Data Availability

The data used to train the models are publicly available at: 10.17863/CAM.107498. The torsion drive data set is also available at: https://zenodo.org/records/11385284. The MACE-OFF series of models is available at: https://github.com/ACEsuit/mace-off/tree/main.

## Notes

Training details for the MACE-OFF models, including filtering of the SPICE data set; detailed breakdown of test set errors, including intramolecular force errors; details of the torsion scans; details of lattice enthalpy calculations; crystal structure geometries; details of condensed-phase simulations, including error tables; details of biological simulations; simulation details and results for dipole models; and computational performance in LAMMPS (PDF)
