# Supplementary material for: MACE-OFF: Short-Range Transferable Machine Learning Force Fields for Organic Molecules
Source: J Am Chem Soc. 2025 May 19;147(21):17598–611. doi: 10.1021/jacs.4c07099 (PMC12123624; doi:10.1021/jacs.4c07099)
Supplement: Supplementary file 1 [file ja4c07099_si_001.pdf]

Supporting Information for: MACE-OFF: Short Range Transferable Machine Learning Force Fields for Organic Molecules

Dávid Péter Kovács*,† J. Harry Moore*,† Nicholas J. Browning,¶ Ilyes Batatia,† Joshua T. Horton,§ Yixuan Pu,‖ Venkat Kapil,⊥ William C. Witt,@ Ioan-Bogdan Magdău,§ Daniel J. Cole,§ and Gábor Csányi*,†

†*Engineering Laboratory, University of Cambridge, Cambridge, CB2 1PZ, UK*

‡*Ångström AI, 2325 3rd Street, San Francisco, CA 94107*

¶*Swiss National Supercomputing Centre (CSCS), 6900, Lugano, Switzerland*

§*School of Natural and Environmental Sciences, Newcastle University, Newcastle upon Tyne NE1 7RU, UK*

‖*Department of Physics and Astronomy, University College, London WC1E 6BT, UK*

⊥*Yusuf Hamied Department of Chemistry, University of Cambridge, Lensfield Road, Cambridge, CB2 1EW,UK*

#*Thomas Young Centre and London Centre for Nanotechnology, London WC1E 6BT, UK*

@*Department of Materials Science and Metallurgy, University of Cambridge, 27 Charles Babbage Road, CB3 0FS, Cambridge, United Kingdom*

E-mail: gc121@cam.ac.uk

S1 MACE-OFF Training Details

Initially, the force weight in the loss was set to 1000 and the energy weight to 40. The learning rate was 0.01 and Adam optimizer with Amsgrad was used. The exponential moving average of the weights was taken in each training step. When the force error converged, the second phase of the training was started with force weight 10 and energy weight 1000 and the learning rate was reduced to 0.00025. Finally, the training was terminated when the energy error also stopped decreasing significantly. All models were trained on a single Nvidia A100 GPU. Training the small model took about 6 days, the medium about 10 days and the large model 14 days.

After fitting the small MACE model and observing the training errors, we noticed the presence of outliers in the dataset. This is probably caused by errors in the underlying electronic structure calculations, some of which have been documented on the SPICE GitHub repository.[1] To confirm this, we also re-evaluated a selection of the configurations that had the highest error, using the level of DFT used in SPICE, and found that for about a third of the configurations, the recomputed energies and forces agreed well with the MACE prediction and not with the original DFT labels. To purify the dataset, we removed from the training set the configurations that had a maximum force error greater than 2 eV/Å. This meant the removal of just 808 configurations, many of which contained heavy elements, in particular phosphorus and iodine, that might have a more challenging electronic structure.

In common with the first version of SPICE, close inspection of the SPICE version 2 dataset revealed several nonphysical geometries with correspondingly high DFT forces. These configurations were removed by applying a maximum force filter of 15 eV/Å. We also observed that many phosphorous-containing compounds were present among the outliers, as well as configurations with highly distorted geometries, which suggest challenging electronic structure. We found that many configurations had forces that do not sum to zero. To avoid

[1] https://github.com/openmm/spice-dataset

the possibility of these configurations poisoning the dataset, we applied a total force filter of 0.1 eV/Å. In total this removed 10,372 configurations from the SPICE version 2 subsets.

S2 Test set errors

The numerical values of the test set errors are displayed in Table S1. Intermolecular force errors were obtained as RMSEs between DFT intermolecular forces and MACE predictions. The intermolecular forces were computed by summing over the translational and rotational components for each atom as shown in Ref.[1]. The algorithm can be summarized as follows:

1. identify molecules (labeled j)

2. within each molecule j sum over all atomic forces (labeled k) to obtain the translational component:
$$F_j^{\text{trans}} = \sum_{k \in j} f_k \tag{1}$$

3. redistribute the molecular translational force onto individual atoms (labeled i) to obtain the atomic translational contributions:
$$f_i^{\text{trans}} = \frac{m_i}{M_j} F_j^{\text{trans}} \tag{2}$$
where m_i are atomic masses and M_j are molecular masses

4. similarly, compute the torque on the entire molecule:
$$T_j = \sum_{k \in j} f_k \times \left(r_k - R_j^{\text{com}}\right) \tag{3}$$

5. compute the rotational atomic force contributions that give rise to the given molecular torque:
$$f_i^{\text{rot}} = m_i \left(r_i - R_j^{\text{com}}\right) \times (I_j^{\alpha\beta})^{-1} T_j \tag{4}$$
where $I_j^{\alpha\beta}$ are the molecular moments of inertia

Table S1: Summary of test set errors including inter-molecular force errors

			PubChem	DES370K Monomers	DES370K Dimers	Dipeptides	Solvated Amino Acids	Water	QMugs	Tripeptide
MAE	E (meV/at)	23S	1.41	1.04	0.98	0.84	1.60	1.67	1.03	1.05
		23M	0.91	0.63	0.58	0.52	1.21	0.76	0.69	0.57
		23L	0.88	0.59	0.54	0.42	0.98	0.83	0.45	0.38
	F_{total} (meV/Å)	23S	35.68	17.63	16.31	25.07	38.56	28.53	41.45	32.88
		23M	20.57	9.36	9.02	14.27	23.26	15.27	23.58	18.74
		23L	14.75	6.58	6.62	10.19	19.43	13.57	16.93	13.20
	F_{inter} (meV/Å)	23S	0.13	0.18	3.01	0.07	16.98	19.03	0.09	0.07
		23M	0.13	0.18	1.79	0.07	10.30	10.03	0.09	0.07
		23L	0.13	0.18	1.44	0.07	8.85	8.97	0.09	0.07
Relative MAE	E (%)	23S	0.00	0.00	0.00	0.00	0.01	8.44	0.00	0.00
		23M	0.00	0.00	0.00	0.00	0.00	3.84	0.00	0.00
		23L	0.00	0.00	0.00	0.00	0.00	4.18	0.00	0.00
	F_{total} (%)	23S	4.65	2.72	3.46	3.55	3.16	4.50	4.12	3.66
		23M	2.68	1.45	1.91	2.02	1.90	2.41	2.35	2.09
		23L	1.92	1.02	1.40	1.44	1.59	2.14	1.68	1.47
	F_{inter} (%)	23S	nan	nan	15.52	nan	21.66	21.19	nan	nan
		23M	nan	nan	9.25	nan	13.14	11.17	nan	nan
		23L	nan	nan	7.44	nan	11.29	9.99	nan	nan
RMSE	E (meV/at)	23S	2.74	1.47	1.51	1.26	1.98	2.07	1.28	1.40
		23M	2.02	0.90	0.91	0.85	1.55	0.99	0.89	0.75
		23L	2.48	0.84	0.87	0.70	1.32	0.99	0.58	0.49
	F_{total} (meV/Å)	23S	61.83	26.15	28.48	36.97	53.55	39.33	62.46	71.41
		23M	40.51	14.31	16.81	22.25	32.19	21.40	36.73	33.94
		23L	33.34	10.27	12.81	16.19	26.91	18.78	27.17	22.46
	F_{inter} (meV/Å)	23S	0.78	0.44	8.70	0.13	27.11	27.59	0.17	0.12
		23M	0.79	0.44	5.54	0.13	16.46	15.05	0.17	0.12
		23L	0.76	0.44	4.34	0.13	14.24	13.39	0.17	0.12
Relative RMSE	E (%)	23S	0.00	0.00	0.00	0.00	0.01	8.30	0.00	0.00
		23M	0.00	0.00	0.00	0.00	0.00	3.96	0.00	0.00
		23L	0.00	0.00	0.00	0.00	0.00	3.98	0.00	0.00
	F_{total} (%)	23S	5.56	2.79	4.13	3.63	3.47	4.58	4.66	5.45
		23M	3.64	1.52	2.44	2.19	2.09	2.49	2.74	2.59
		23L	3.00	1.09	1.86	1.59	1.74	2.19	2.03	1.71
	F_{inter} (%)	23S	nan	nan	8.95	nan	20.17	20.18	nan	nan
		23M	nan	nan	5.70	nan	12.25	11.00	nan	nan
		23L	nan	nan	4.46	nan	10.60	9.79	nan	nan

6. compute the vibrational contribution as the difference:

$$f_i^{\text{vib}} = f_i - f_i^{\text{trans}} - f_i^{\text{rot}} \tag{5}$$

7. the intermolecular force is calculated as the sum of the translational and rotational contributions.

Relative errors were obtained by dividing the absolute errors by the typical DFT force magnitudes:

$$\text{Rel MAE} = \frac{\sum_i |f_i^{DFT} - f_i^{MACE}|}{\sum_i |f_i^{DFT} - \bar{f}^{DFT}|} \tag{6}$$

$$\text{Rel RMSE} = \sqrt{\frac{\sum_i (f_i^{DFT} - f_i^{MACE})^2}{\sum_i (f_i^{DFT} - \bar{f}^{DFT})^2}} \tag{7}$$

where $\bar{f}^{DFT} = \sum_i f_i^{DFT}/\text{N}$ is the average DFT force (often zero).

Table S1 shows these errors computed for all three MACE models on each category of atomic configurations found in the test set. Interestingly, even though PubChem, DES370K Monomers, Dipeptides, QMugs and Tripeptides comprise only isolated molecule configurations, we find a small error that is incorrectly attributed to intermolecular interactions. This issue arises because DFT forces in these test data to not obey translational and rotational symmetry, i.e. total molecular force and torque do not sum to zero, even though a single molecule is present in each simulation box. Meanwhile, MACE forces do obey these symmetries, and therefore the difference shows up as an intermolecular error which is identical for all three MACE models.

The code used for this analysis is available at `https://github.com/imagdau/aseMolec.git`.

S2.1 MACE-OFF24(M) Test Errors

Table S2: Test set errors for MACE-OFF24(M)

	MAE Energy (meV/atom)	MAE Force (meV/Å)
PubChem	1.0	22.1
DES370K Monomers	0.6	9.6
DES370K Dimers	0.6	9.3
Dipeptides	0.5	14.4
QMugs	0.8	23.8
Solvated Amino Acids	1.3	23.2
Amino Acid-Ligand	1.5	24.2
Solvated PubChem	1.2	22.8

S3 Torsion angle scanning calculations

The TorsionNet-500[2] and biaryl torsion[3] benchmark datasets were recomputed at the SPICE[4] level of QM theory (ωB97M-D3(BJ)/def2-TZVPPD[5–9]) to assess the accuracy of the MACE models in predicting the potential energy surfaces of rotatable bonds commonly found in drug-like molecules. Starting from the published optimized geometries, a torsion scan was performed using TorsionDrive[10] via its interface with QCEngine[11]. The dihedral angles were scanned in 15° increments and each constrained geometry optimization was carried out with geomeTRIC[12], using PSI4[13] to calculate the DFT energies and forces. For each of the assessed force field models, a separate constrained geometry optimization was performed starting from the DFT reference geometries and holding the target torsion fixed at each grid point value. The final RMSD between the optimized and reference geometry was recorded, along with the energy to calculate the potential energy surfaces.

S4 Lattice enthalpies

First, the internal energy of the crystalline form was computed under the harmonic approximation:

$$\begin{aligned} U(T) &= \left(\frac{\partial \ln Z}{\partial \frac{1}{k_B T}}\right) \\ &= E_{\mathrm{MACE}} + \int_0^\infty \left[\frac{\epsilon}{e^{\frac{\epsilon}{k_B T}} - 1} + \frac{\epsilon}{2}\right] \sigma(\epsilon)\mathrm{d}\epsilon \end{aligned} \tag{8}$$

where Z denotes the partition function of the system, and $\sigma(\epsilon)$ represents the degeneracy or phonon density of states as a function of vibrational energy. The above expression contains an electronic contribution, computed using MACE, a zero point energy contribution, and a contribution from finite-temperature vibrational energy. To compute the vibrational contribution, we used a $3 \times 3 \times 3$ supercell for all the molecules considered. For the gaseous form of the molecules, we employed the ideal gas approximation to compute the enthalpies of the gas as:

$$U(T) = e_{\mathrm{MACE}} + E_{\mathrm{ZPE}} + \int_0^T C_p \mathrm{d}T \tag{9}$$

where we used the ideal gas value to evaluate the last term, giving $4RT$ for all system except for the linear carbon-dioxide, where the value is $3.5RT$.

Combining the above two expressions, we can compute the sublimation enthalpies as:

$$\begin{aligned} \Delta H_{\mathrm{subm}} &= U_{\mathrm{gas}}(T) - U_{\mathrm{cryst}}(T) \\ &= E_{\mathrm{latt}} + \Delta E_{\mathrm{vib}} + 4RT \end{aligned} \tag{10}$$

To assess the accuracy of the MACE model we used the X23 set of molecular crystals[14]. We first relaxed the cells, followed by phonon and normal mode calculations to obtain the finite temperature vibrational as well as zero point contributions to the enthalpies, following the protocol above. It is important to note that for the ANI-2x force field it was not possible

to fully relax the crystal geometries, because the model cannot predict stresses. Hence, we kept the cell fixed at the DFT equilibrium values from Ref.[14]. The results are summarized in Table S3.

Table S3: **Sublimation enthalphy in kJ/mol**

System	ΔH^0_{sub} exp.	ΔH^0_{sub} MACE-OFF23(S)	ΔH^0_{sub} MACE-OFF23(M)	ΔH^0_{sub} MACE-OFF23(L)	ΔH^0_{sub} ANI-2x
1,4-Cyclohexanedione	81.1	85.1	77.7	85.1	59.7
Acetic acid	67.7	77.2	71.4	70.3	90.7
Adamantane	61.6	88.7	65.6	69.2	67.1
Ammonia	31.2	24.4	30.9	26.8	23.0
Anthracene	101.9	118.8	95.7	106.3	98.9
Benzene	44.9	49.4	48.1	58.4	48.3
Carbon dioxide	26.1	-35.2	-1.44	4.1	-117.9
Cyanamide	75.5	85.8	94.5	87.3	36.9
Cytosine	156.4	159.8	142.8	143.2	118.6
Ethyl carbamate	78.8	88.7	90.9	91.4	77.0
Formamide	71.7	82.2	75.0	80.1	67.3
Hexamine	75.8	58.4	89.5	62.3	83.5
Imidazole	81.4	88.7	91.6	86.3	86.6
Naphthalene	72.6	88.9	80.1	83.6	72.4
Oxalic acid α	93.7	98.5	89.5	89.7	44.1
Oxalic acid β	93.6	90.0	95.2	93.5	45.4
Pyrazine	56.3	86.4	70.0	69.7	53.7
Pyrazole	72.4	67.5	78.6	73.0	75.4
s-Triazine	55.7	58.4	64.9	75.6	112.1
s-Trioxane	56.3	75.8	59.9	53.9	51.7
Succinic acid	123.1	132.5	119.7	123.5	141.7
Uracil	129.2	139.7	127.2	131.8	98.9
Urea	93.8	121.5	114.0	112.5	78.2

S5 Crystal structure geometries

In Table S4 we compare the MACE-OFF23(L) relaxed crystal structure lattice parameters with the experimental ones reported in Ref.[15]. We confirm that MACE is able to accurately reproduce the lattice constant of molecular crystals purely trained on molecular dimers. Additional trimer training data, or a higher level of reference theory, such as coupled cluster, could probably further improve the agreement with experiments.

Table S4: **Crystal structure geometries**

Molecule	T_{exp}		Experiment	MACE-OFF23(L)
Acetic acid	40	a	13.151	13.359
		b	3.923	3.809
		c	5.762	5.542
		V	297.27	282.01
Ammonia	2	a	5.048	4.946
		V	128.63	120.98
Benzene	4	a	7.351	6.59
		b	9.364	9.303
		c	6.695	6.851
		V	460.84	420.51
Naphthalene	10	a	8.0846	7.821
		b	5.9375	5.836
		c	8.6335	8.430
		β	124.67	125.26
		V	340.83	314.23
Pyrazine	184	a	9.325	9.351
		b	5.850	5.508
		c	3.733	3.545
		V	203.64	182.57
Urea	40	a	5.565	5.330
		c	4.684	4.670
		V	145.06	123.68

S6 Condensed phase simulations

Water density simulations were seeded using OpenMM's Modeller functionality, with a padding of 12.5 Å from the central water molecule to the box edge, resulting in box edge of 25 Å. Simulations were run for 500 000 steps with a 1 fs timestep, resulting in a 500 ps trajectory. The density was recorded every 100 steps. After an initial 100 ps period of equilibration, the density was calculated by averaging over the remaining 400 ps of trajectory.

Initial PDB files for the remaining organic liquid simulations were taken from Ref.[16] and periodic boxes were generated using packmol[17]. Boxes were prepared containing 64 molecules, with box vectors determined such that the initial density was 80% of the experimental density. Structures were minimized with the L-BFGS algorithm prior to MD. Molecular dynamics was performed with OpenMM, using a custom fork of the `openmm-ml` package to interface MACE models to the MD code.[2] Simulations were performed in the NPT ensemble. The Langevin equations of motion were integrated with a timestep of 1 fs and a Monte Carlo barostat was used to maintain pressure at 1 atm. Temperatures were maintained at 298 K except for those compounds whose boiling point was below this value, for which a value of 10 K below the experimental boiling point was used. Dynamics were propagated for 300 ps, and the final density was calculated by averaging over the final 100 ps of the simulation.

Heat of vaporization calculations additionally required simulation of the isolated molecule in the vacuum phase, which were carried out on a nonperiodic molecule. Final energies for both condensed and vacuum phases were calculated by averaging over the final 100 ps of each trajectory.

Tables S5 and S6 summarize the overall density and heat of vaporization errors for the MACE-OFF23(S) and MACE-OFF23(M) models. Although the computational expense of the MACE-OFF23(L) is prohibitive for these condensed phase simulations, we nonetheless

[2] https://github.com/jharrymoore/openmm-ml/tree/main

benchmarked the large model on a small selection of condensed phase systems. We saw comparable performance to the medium model, and in particular observed that compounds that had a high prediction error under the medium model had a similar error under the large model. This indicates that this error is driven primarily by the dataset rather than by the size of the model, as previously hypothesized.

To investigate this further, Tables S5 and S6 additionally show statistics for MACE-OFF24(M), which employs an extended cutoff of 6 Å and has also been trained on additional nonbonded data. We observe a modest decrease in MAE for the heat of vaporization from 2.18 to 1.75 kcal/mol, as well as a small decrease in the accuracy of liquid density predictions. This further reinforces the hypothesis that the poorly predicted densities are a result of under-representation of the functional groups, since the additional SPICE training sets do little to increase sampling of these problematic functional groups.

Table S5: **Summary of density errors**

	MAE / g/cm^3	RMSE / g/cm^3	r
MACE-OFF23(S)	0.23	0.38	0.20
MACE-OFF23(M)	0.09	0.15	0.89
MACE-OFF24(M)	0.14	0.34	0.26
ANI-2x	0.21	0.36	0.26

Table S6: **Summary of ΔH_{vap} errors**

	MAE / kcal/mol	RMSE / kcal/mol	r
MACE-OFF23(S)	1.48	2.19	0.86
MACE-OFF23(M)	2.18	2.53	0.87
MACE-OFF24(M)	1.75	2.81	0.78
ANI-2x	2.75	3.45	0.66

S7 Biological Simulations

S7.1 Ala_3 Free Energy Surface

Molecular dynamics simulations were performed with OpenMM, as in the previous section. Enhanced sampling simulations were performed with metadynamics. Two collective variables were defined by the two torsion angles on the backbone of the central alanine residue. A 1 ns simulation was performed with a temperature bias factor of 10, a barrier height of 1 kJ/mol and a frequency of 100 steps.

S7.2 Ala_{15} peptide folding

The initial extended structure was generated using PyMOL. The system was propagated with Langevin dybamics using a 1 fs timestep for 500 ps *in vacuo* via the OpenMM interface.

S7.2.1 Secondary structure prediction with MACE-OFF23(M)

Compared to MACE-OFF24(M) (see main text), the previous 5 Å model failed to reproduce the experimentally observed oscillation between the α-helix and the 3_{10} helix, instead predicting only the α-helical structure

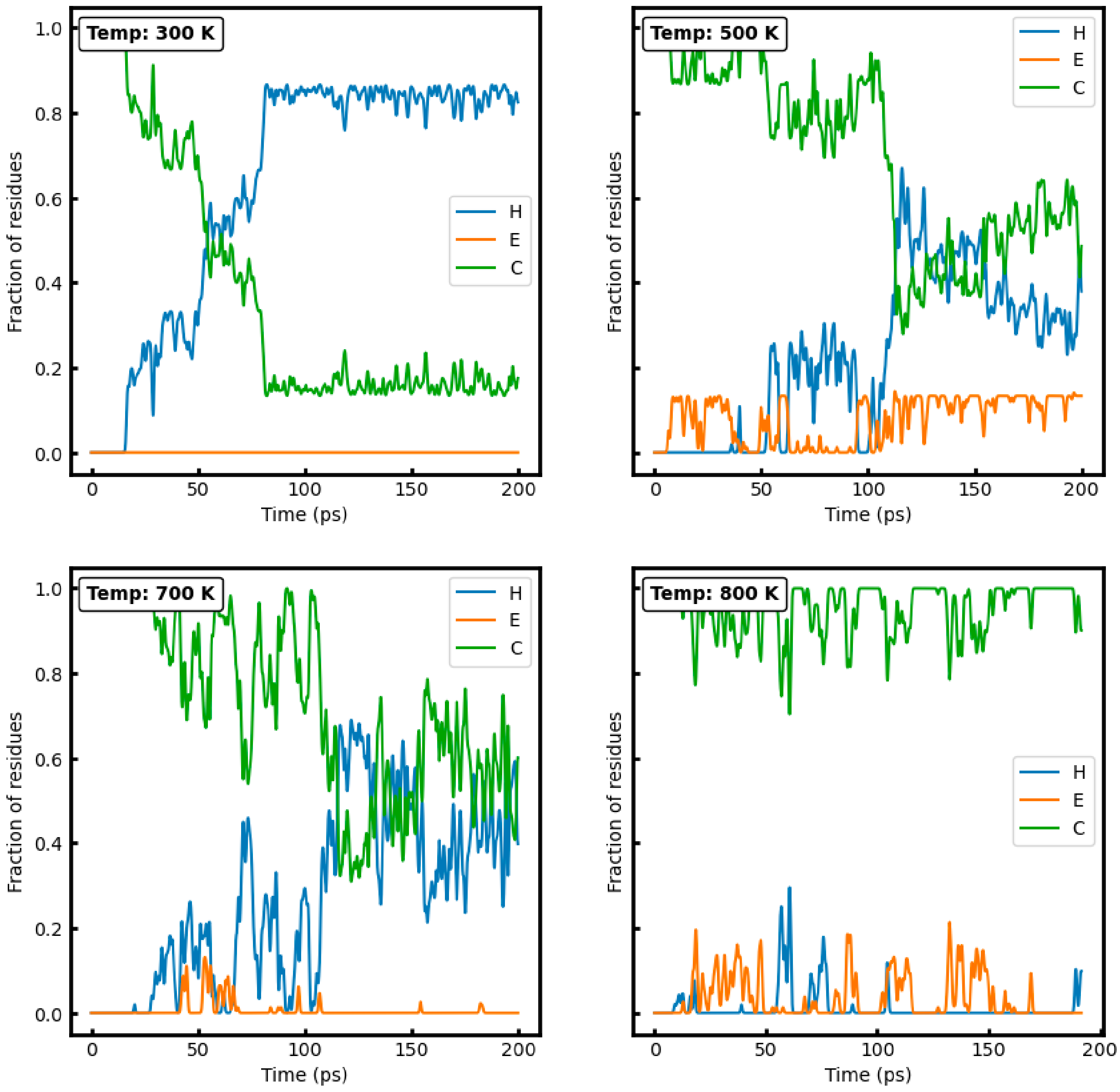

Figure S1: Secondary structure (and temperature dependence) of Ala_{15} predicted by MACE-OFF23(M). Single letter codes correspond to standard STRIDE secondary structure labels, where H=α-helix, E=extended and C=random coil

S7.3 Crambin in explicit solvent

Crambin was prepared from the PDB structure 1EJG. The initial structure was prepared using pdbfixer[18] and solvated in an orthorombic box with padding between solute and the box edge set to 1.2 nm. Dynamics were performed in the NPT emsemble, with pressure

maintained at 1 atm with a Monte Carlo barostat, as implemented in OpenMM. A 1 fs timestep was used both for integrating the Langevin equations of motion and for writing the trajectory to disk for post processing. The power spectrum was calculated as the Fourier transform of the velocity autocorrelation function using the Travis program with default settings[19–22].

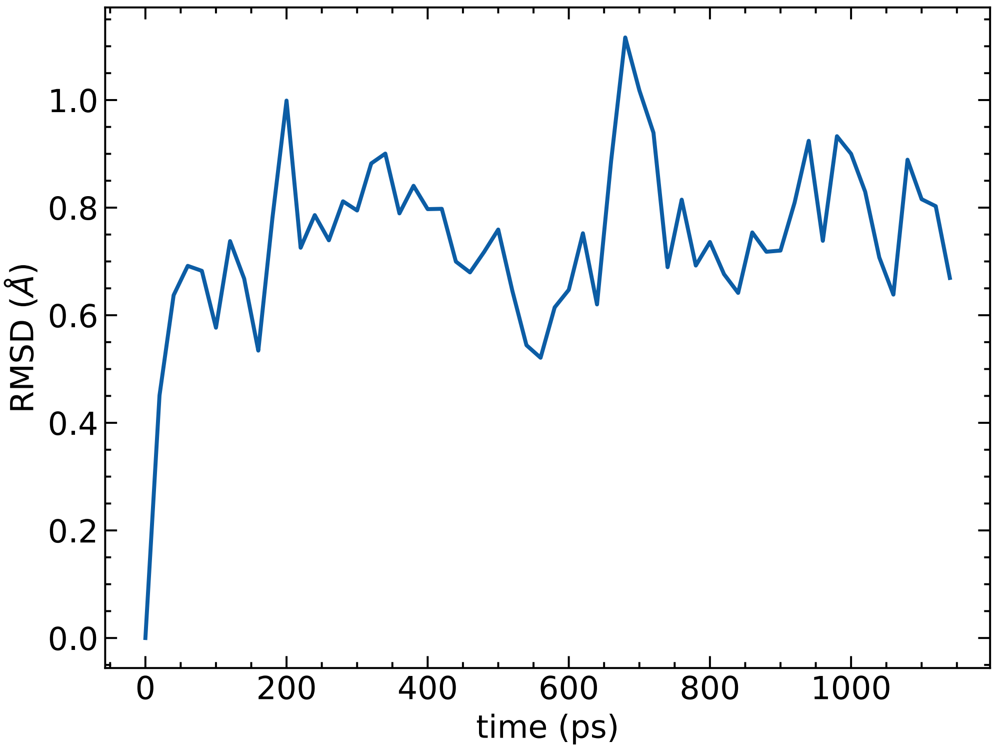

Figure S2: **Crambin RMSD.** RMSD calculated by averaging two halves of a 1 ns trajectory, relative to the first frame and computed on C_α atoms.

S8 Dipole models

S8.1 MACE-OFF23-μ

In addition to the series of MACE-OFF force field models, we also developed MACE-OFF23-μ, which predicts the total dipole moment of molecules and can be used, in combination with a regular MACE-OFF model for energies and forces, to estimate vibrational spectra based on the autocorrelation of the dipole moment. The model architecture is identical to the MACE architecture described in the Methods in the main text, with the only difference being the readout function, which produces an $L = 1$ tensor for each atom instead of the atomic site energies. Given the equivariant order of the readout, the smallest possible model corresponds to the 'medium' architecture, which is the one considered in this work. Other than the readout's equivariant order, all hyperparameters match those of the MACE-OFF23(M) model. MACE-OFF23-μ was trained on the subset of SPICE[4] for which a total dipole moment was available, resulting in the exclusion of the QMugs and water subsets.

We assess the accuracy of the MACE-OFF23-μ model on the held-out SPICE test sets, evaluating its effectiveness for predicting dipole moments and infrared spectra of organic molecules. Figure S3 summarizes this performance by examining the alignment between the violin distributions of the reference and predicted total dipole moment values, as well as the RMSEs. The small RMSEs and strong alignment of the distributions indicate high fidelity across the test datasets for which reference dipole moment values were available. These results suggest promising performance for zero-shot predictions of the dipole moments (and infrared spectra) of unseen organic molecules, as demonstrated in a recent benchmark on out-of-distribution drug-like molecules from the NIST database[23]. The MACE-OFF23-μ model is available at: `https://github.com/venkatkapil24/MACE-OFF-mu-models`.

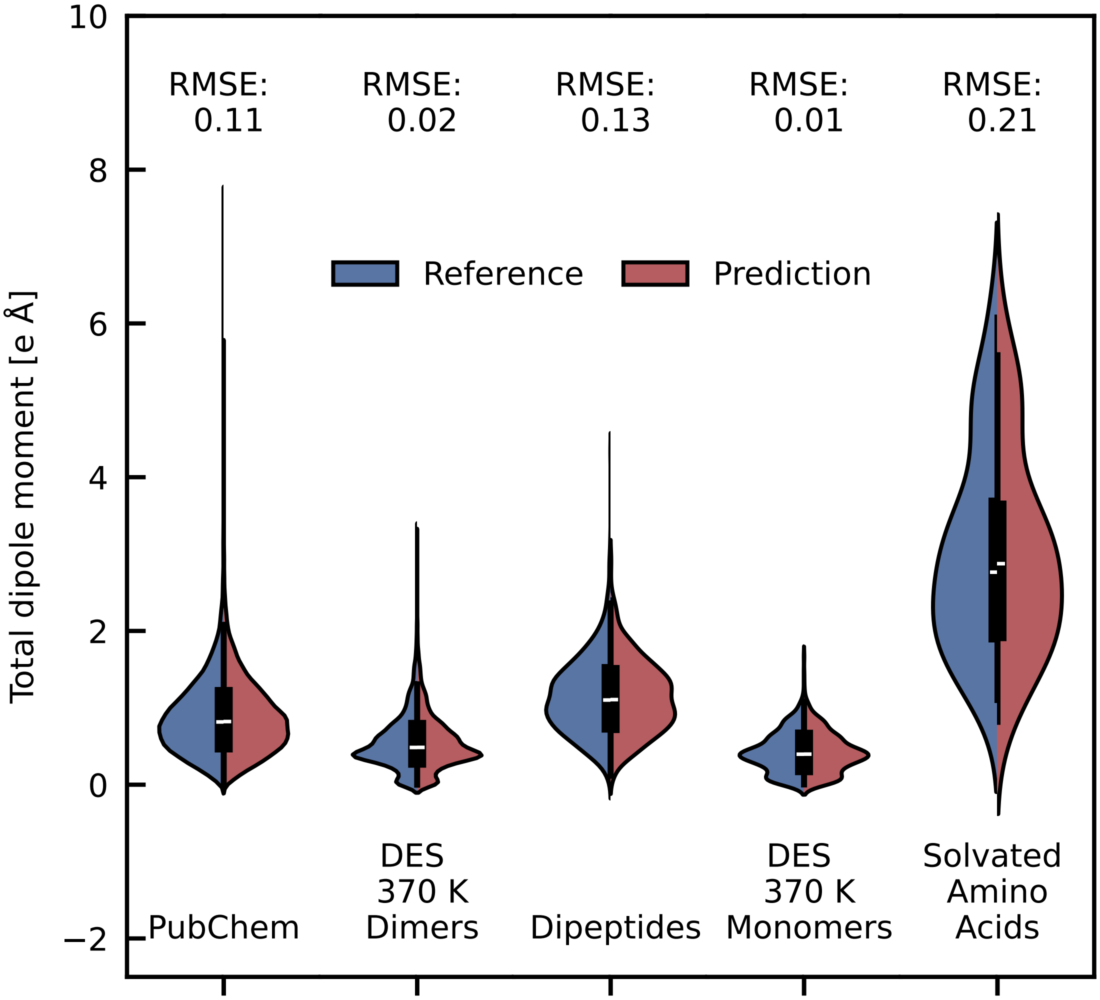

Figure S3: **Test set prediction and root mean square errors (RMSE).** Violin plots of the reference (blue) and predicted (red) distributions of the dipole moment predicted using MACE-OFF23-μ. The RMSEs are reported above the violin plots for the individual test sets.

S8.2 Computational details

To compute the vibrational spectrum for the paracetamol form II polymorph, we first performed a 20 ps path-integral MD simulation using the MACE-OFF23(S) model at 500 K, following the prescriptions of the Path Integral coarse-Grained Simulations (PIGS) approach[24,25]. The path-integral MD simulations are performed with a 0.50 fs timestep using the BAOAB integration scheme extended to path-integral MD[26] using the path-integral Langevin equation thermostats[27] with a time constant of 100 fs.

To generate a potential energy surface that effectively encodes quantum nuclear effects, we followed the PIGS technique. We used the positions and forces on the centroid of the path-integral to fit a MACE model that represents the difference between the potential energy surface and the potential of mean force on the centroid at an elevated temperature of 500 K. The sum of MACE-OFF23(S) and the PIGS model gives a transferable effective potential energy surface that encodes quantum nuclear effects.

To generate a trajectory with quantum nuclear motion, we run MD with the sum of MACE-OFF23(S) and the PIGS model using the `i-PI`[28] software to propagate the equations of motion and the `ASE` calculator as the force-provider. The MD simulations were performed with a 0.50 fs timestep using a weak global stochastic velocity rescaling thermostat. To generate classical trajectories we perform MD with the same setup but without the PIGS potential.

We predicted the $L = 0$ and $L = 2$ spherical harmonic components of polarizability tensors on the trajectories and computed their time correlation functions to obtain the isotropic and anisotropic Raman spectra[24]. We estimate the powder Raman spectrum as a linear combination of the isotropic and the anisotropic Raman spectra[29].

The model architecture was identical to the MACE architecture used for the energy models, with the only difference being the readout function producing a vector and a tensor (dipole and polarisability) for each atom, instead of the atomic site energies. A relatively

small MACE model is already capable of achieving high accuracy predictions, and the selected models used the hyperparameters displayed in Table S7.

Table S7: Hyperparameters of the MACE models for dipoles and polarizabilities

	Paracetamol	Water
Cutoff radius (Å)	5.0	6.0
Chemical channels k (Eq. 1)	16	32
max L (Eq. 5)	2	2

S8.3 Vibrational spectroscopy of paracetamol

Raman spectroscopy is one of the most widely used techniques for characterizing molecular crystals. Unlike IR spectroscopy, which only detects vibrational modes that distort dipoles, Raman spectroscopy is more sensitive to collective modes governed by weak, non-bonded interactions in a broad range of molecular materials. The low-frequency region of the Raman spectrum (e.g., the THz regime) gives a vibrational fingerprint of the intermolecular interactions. Thus, it is widely used to differentiate between polymorphs of molecular crystals. Meanwhile, the high-frequency Raman spectrum probes intramolecular modes and their coupling to low-frequency modes.

Here, we test the ability of the MACE-OFF23(S) model to predict the Raman spectrum of the "Form II" polymorph of paracetamol. To compare MACE-OFF23(S) directly with experiments, we rigorously incorporate quantum nuclear and non-Condon effects using our ML-aided framework[24]. We incorporate quantum nuclear effects by fitting an effective potential energy surface[25] (using MACE) to calculate quantum nuclear corrections to the MACE-OFF23(S) model within the path-integral coarse-grained simulations (PIGS) method. To incorporate non-Condon effects, we fit a separate equivariant MACE model to the first-principles polarizability of paracetamol polymorphs (data taken from Refs. 30,31). The

remaining steps of our quantum nuclear simulations mimic an entirely classical calculation involving a NVE molecular dynamics simulation, prediction of the isotropic and anisotropic components of the polarizability tensor, and calculation of their time correlation functions. For further details we refer the reader to Ref. 24.

As shown in Figure S4, we predict both the high- and low-frequency regions of the Raman spectrum of paracetamol form II with an overall good agreement with the experimental band positions[32]. Since the experiment captures the Raman spectra along different crystal directions while we estimate the "powder" Raman spectrum[31], we do not compare band intensities. We find that the classical predictions (based on MACE classical MD) are consistently shifted with respect to the experiment. At the same time, quantum nuclear predictions (encoded by the PIGS method) play an essential role in improving the agreement between theory and experiments. Moreover, we note that a broad band at around 3300 cm^{-1} is only captured at the level incorporating quantum nuclear effects. The low-frequency modes also agree semi-quantitatively with the experiments and do not require a quantum nuclear description. The 2–3 % overall shift in the vibrational frequencies notwithstanding, the MACE-OFF23(S) combined with quantum nuclear effects and non-Condon effects shows promising capabilities for the characterization of molecular crystals.

S8.4 Vibrational spectroscopy of liquid water

Next, we characterize the dynamical properties of water by computing the vibrational density of states, including the IR and Raman spectra selection rules, using MACE models of dipoles and polarizabilities[24]. Given the significant impact of quantum nuclear motion on the dynamics of water[25], we incorporate quantum nuclear effects using the PIGS approach[25] (discussed in Section S8.3), which has a classical cost and has recently been shown to describe quantum nuclear effects in water accurately[34]. As shown in Figure S5, after incorporating quantum nuclear effects, the MACE-OFF23(S) predictions agree with the ex-

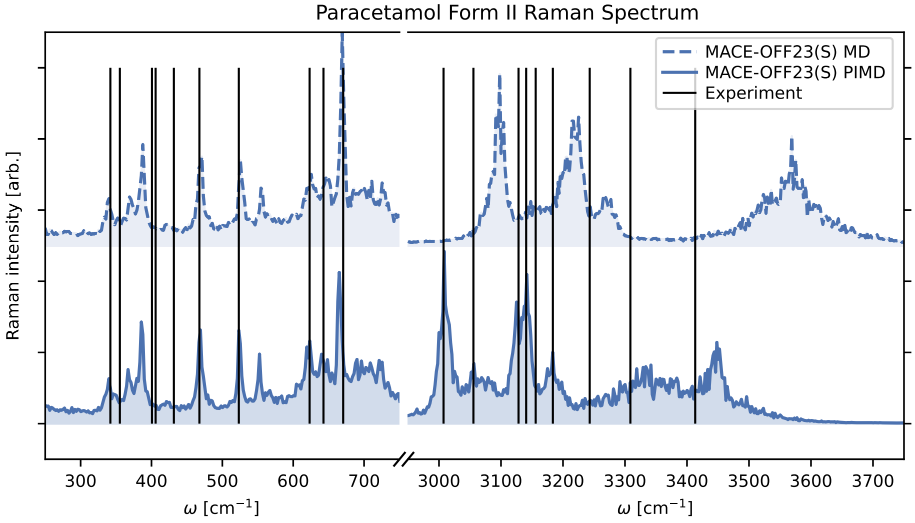

Figure S4: **Powder Raman spectrum of paracetamol form II[33].** Spectrum computed at ambient conditions using the MACE-OFF23(S) model for the potential energy surface, a MACE model of the polarizability, and a MACE model that incorporates quantum nuclear effects on the potential energy surface using the PIGS approach[25]. The black lines represent experimentally determined band positions[32], scaled by 3% to aid visual comparison with the predicted spectra. We focus on high and low frequency features of the spectrum where experimental peaks are well separated.

periments across the entire frequency range. We note the presence of an overall 2-3 % blue shift with respect to the experiments, consistent with the spectra for paracetamol form II (see Section S8.3). MACE-OFF23(S) even qualitatively captures subtle features of the spectra, including the 0 – 1000 cm^{-1} hydrogen bonding fingerprints in the anisotropic Raman spectrum, and the bimodal nature of the isotropic Raman stretching band arising from the presence of hydrogen-bonded defects in room temperature water.

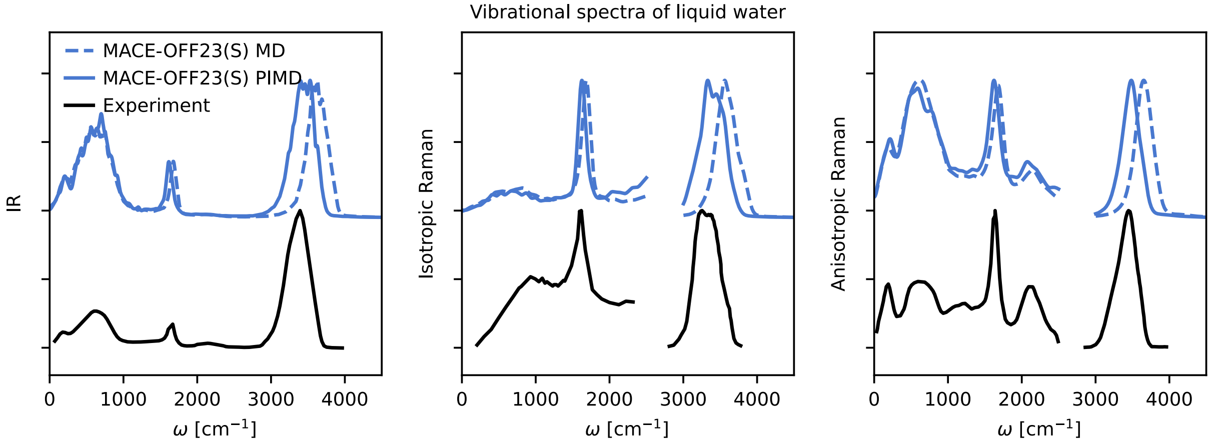

Figure S5: IR, isotropic Raman and anisotropic Raman spectra of water at ambient temperature and density using the MACE-OFF23(S) model for the potential energy surface and a single MACE model of water's dipole and polarizability[24]. The black curve represents the experiment[35]. The dashed blue curve is obtained from classical MD, while the solid curve incorporates quantum effects using the PIGS method[25].

S9 Computational performance in LAMMPS

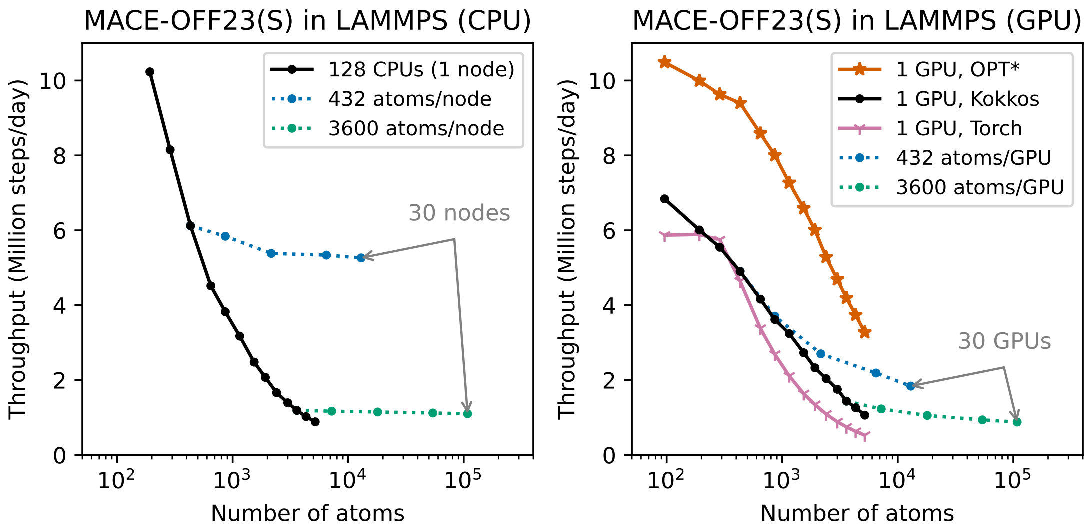

Figure S6: **Throughput in LAMMPS.** Performance of molecular dynamics in LAMMPS for water in the NVT ensemble at 1 g/cm^3 and 300 K with a 1 fs timestep. All simulations used variants of the MACE-OFF23(S) model.

The left panel highlights CPU performance achievable on the UK-based Archer2 CPU supercomputer, using the new pure-C++ MACE implementation. The solid black curve demonstrates the throughput of a single 128-core node as a function of the number of atoms. The two dotted curves demonstrate weak scaling with 432 atoms/node or 3600 atoms/node. All calculations used double precision floating point numbers.

The right panel demonstrates the performance of the GPU evaluators. The three solid curves provide single-GPU results (determined with NVIDIA A100 80GB GPUs) for the native Torch-based MACE-OFF23(S), a custom Kokkos-based MACE implementation, and the `cuda_mace` implementation marked as OPT*. The asterisk indicates that these latter simulations used single precision floating point numbers, with error correction for the most numerically sensitive terms, in contrast to the remaining calculations which were performed with double precision. Finally, the two dashed curves demonstrate the performance when multiple GPUs are employed, using the pure-Kokkos implementation, to access larger systems with domain decomposition. More work is needed to improve this weak scaling.

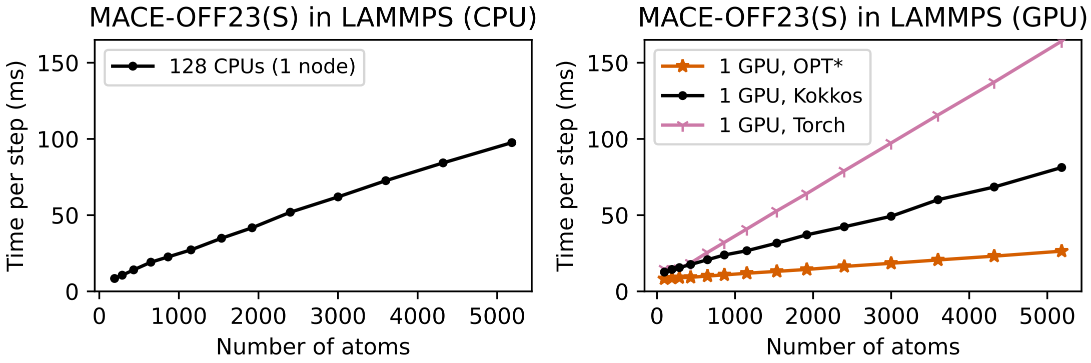

Figure S7: **Evaluation speed in LAMMPS.** Data from Figure S6, replotted to show the average duration of each timestep.

References

(1) Magdău, I.-B.; Arismendi-Arrieta, D. J.; Smith, H. E.; Grey, C. P.; Hermansson, K.; Csányi, G. Machine learning force fields for molecular liquids: Ethylene Carbonate/Ethyl Methyl Carbonate binary solvent. *npj Computational Materials* **2023**, *9*, 146.

(2) Rai, B. K.; Sresht, V.; Yang, Q.; Unwalla, R.; Tu, M.; Mathiowetz, A. M.; Bakken, G. A. Torsionnet: A deep neural network to rapidly predict small-molecule torsional energy profiles with the accuracy of quantum mechanics. *Journal of Chemical Information and Modeling* **2022**, *62*, 785–800.

(3) Lahey, S.-L. J.; Thien Phuc, T. N.; Rowley, C. N. Benchmarking force field and the ani neural network potentials for the torsional potential energy surface of biaryl drug fragments. *Journal of Chemical Information and Modeling* **2020**, *60*, 6258–6268.

(4) Eastman, P.; Behara, P. K.; Dotson, D. L.; Galvelis, R.; Herr, J. E.; Horton, J. T.; Mao, Y.; Chodera, J. D.; Pritchard, B. P.; Wang, Y.; others Spice, a dataset of drug-like molecules and peptides for training machine learning potentials. *Scientific Data* **2023**, *10*, 11.

(5) Najibi, A.; Goerigk, L. The nonlocal kernel in van der Waals density functionals as an additive correction: An extensive analysis with special emphasis on the B97M-V and ωB97M-V approaches. *Journal of Chemical Theory and Computation* **2018**, *14*, 5725–5738.

(6) Weigend, F.; Ahlrichs, R. Balanced basis sets of split valence, triple zeta valence and quadruple zeta valence quality for H to Rn: Design and assessment of accuracy. *Physical Chemistry Chemical Physics* **2005**, *7*, 3297–3305.

(7) Rappoport, D.; Furche, F. Property-optimized Gaussian basis sets for molecular response calculations. *The Journal of chemical physics* **2010**, *133*.

(8) Grimme, S.; Ehrlich, S.; Goerigk, L. Effect of the damping function in dispersion corrected density functional theory. *Journal of computational chemistry* **2011**, *32*, 1456–1465.

(9) Grimme, S.; Antony, J.; Ehrlich, S.; Krieg, H. A consistent and accurate ab initio parametrization of density functional dispersion correction (DFT-D) for the 94 elements H-Pu. *The Journal of chemical physics* **2010**, *132*.

(10) Qiu, Y.; Smith, D. G.; Stern, C. D.; Feng, M.; Jang, H.; Wang, L.-P. Driving torsion scans with wavefront propagation. *The Journal of chemical physics* **2020**, *152*.

(11) Smith, D. G.; Lolinco, A. T.; Glick, Z. L.; Lee, J.; Alenaizan, A.; Barnes, T. A.; Borca, C. H.; Di Remigio, R.; Dotson, D. L.; Ehlert, S.; others Quantum chemistry common driver and databases (QCDB) and quantum chemistry engine (QCEngine): Automation and interoperability among computational chemistry programs. *The Journal of chemical physics* **2021**, *155*.

(12) Wang, L.-P.; Song, C. Geometry optimization made simple with translation and rotation coordinates. *The Journal of chemical physics* **2016**, *144*.

(13) Smith, D. G.; Burns, L. A.; Simmonett, A. C.; Parrish, R. M.; Schieber, M. C.; Galvelis, R.; Kraus, P.; Kruse, H.; Di Remigio, R.; Alenaizan, A.; others PSI4 1.4: Open-source software for high-throughput quantum chemistry. *The Journal of chemical physics* **2020**, *152*.

(14) Reilly, A. M.; Tkatchenko, A. Understanding the role of vibrations, exact exchange, and many-body van der Waals interactions in the cohesive properties of molecular crystals. *The Journal of chemical physics* **2013**, *139*, 024705.

(15) Dolgonos, G. A.; Hoja, J.; Boese, A. D. Revised values for the X23 benchmark set of molecular crystals. *Physical Chemistry Chemical Physics* **2019**, *21*, 24333–24344.

(16) Horton, J. T.; Allen, A. E.; Dodda, L. S.; Cole, D. J. QUBEKit: Automating the derivation of force field parameters from quantum mechanics. *Journal of chemical information and modeling* **2019**, *59*, 1366–1381.

(17) Martínez, L.; Andrade, R.; Birgin, E. G.; Martínez, J. M. PACKMOL: A package for building initial configurations for molecular dynamics simulations. *Journal of Computational Chemistry* **2009**, *30*, 2157–2164.

(18) Eastman, P.; Swails, J.; Chodera, J. D.; McGibbon, R. T.; Zhao, Y.; Beauchamp, K. A.; Wang, L.-P.; Simmonett, A. C.; Harrigan, M. P.; Stern, C. D.; Wiewiora, R. P.; Brooks, B. R.; Pande, V. S. OpenMM 7: Rapid Development of High Performance Algorithms for Molecular Dynamics. *PLOS Computational Biology* **2017**, *13*, e1005659.

(19) Brehm, M.; Kirchner, B. TRAVIS - A Free Analyzer and Visualizer for Monte Carlo and Molecular Dynamics Trajectories. *Journal of Chemical Information and Modeling* **2011**, *51*, 2007–2023.

(20) Brehm, M.; Thomas, M.; Gehrke, S.; Kirchner, B. TRAVIS—A Free Analyzer for Trajectories from Molecular Simulation. *The Journal of Chemical Physics* **2020**, *152*, 164105.

(21) Thomas, M.; Brehm, M.; Fligg, R.; Vöhringer, P.; Kirchner, B. Computing Vibrational Spectra from Ab Initio Molecular Dynamics. *Physical Chemistry Chemical Physics* **2013**, *15*, 6608–6622.

(22) Thomas, M.; Brehm, M.; Kirchner, B. Voronoi Dipole Moments for the Simulation of Bulk Phase Vibrational Spectra. *Physical Chemistry Chemical Physics* **2015**, *17*, 3207–3213.

(23) Pracht, P.; Pillai, Y.; Kapil, V.; Csányi, G.; Gönnheimer, N.; Vondrák, M.; Margraf, J. T.; Wales, D. J. Efficient Composite Infrared Spectroscopy: Combining the

Double-Harmonic Approximation with Machine Learning Potentials. *Journal of Chemical Theory and Computation* **2024**, *20*, 10986–11004.

(24) Kapil, V.; Kovács, D. P.; Csányi, G.; Michaelides, A. First-principles spectroscopy of aqueous interfaces using machine-learned electronic and quantum nuclear effects. *Faraday Discussions* **2023**,

(25) Musil, F.; Zaporozhets, I.; Noé, F.; Clementi, C.; Kapil, V. Quantum dynamics using path integral coarse-graining. *The Journal of Chemical Physics* **2022**, *157*.

(26) Kapil, V.; Wieme, J.; Vandenbrande, S.; Lamaire, A.; Van Speybroeck, V.; Ceriotti, M. Modeling the Structural and Thermal Properties of Loaded Metal–Organic Frameworks. An Interplay of Quantum and Anharmonic Fluctuations. *Journal of Chemical Theory and Computation* **2019**, *15*, 3237–3249.

(27) Ceriotti, M.; Parrinello, M.; Markland, T. E.; Manolopoulos, D. E. Efficient stochastic thermostatting of path integral molecular dynamics. *The Journal of Chemical Physics* **2010**, *133*.

(28) Kapil, V. et al. i-PI 2.0: A universal force engine for advanced molecular simulations. *Computer Physics Communications* **2019**, *236*, 214–223.

(29) Raimbault, N.; Athavale, V.; Rossi, M. Anharmonic effects in the low-frequency vibrational modes of aspirin and paracetamol crystals. *Physical Review Materials* **2019**, *3*, 053605.

(30) Raimbault, N.; Grisafi, A.; Ceriotti, M.; Rossi, M. Using Gaussian process regression to simulate the vibrational Raman spectra of molecular crystals. *New Journal of Physics* **2019**, *21*, 105001.

(31) Raimbault, N.; Grisafi, A.; Ceriotti, M.; Rossi, M. Using Gaussian process regression to

simulate the vibrational Raman spectra of molecular crystals. *New Journal of Physics* **2019**, *21*, 105001.

(32) Kolesov, B. A.; Mikhailenko, M. A.; Boldyreva, E. V. Dynamics of the intermolecular hydrogen bonds in the polymorphs of paracetamol in relation to crystal packing and conformational transitions: a variable-temperature polarized Raman spectroscopy study. *Physical Chemistry Chemical Physics* **2011**, *13*, 14243.

(33) Raimbault, N.; Athavale, V.; Rossi, M. Anharmonic effects in the low-frequency vibrational modes of aspirin and paracetamol crystals. *Physical Review Materials* **2019**, *3*.

(34) Ceriotti, M.; Fang, W.; Kusalik, P. G.; McKenzie, R. H.; Michaelides, A.; Morales, M. A.; Markland, T. E. Nuclear Quantum Effects in Water and Aqueous Systems: Experiment, Theory, and Current Challenges. *Chemical Reviews* **2016**, *116*, 7529–7550.

(35) Morawietz, T.; Marsalek, O.; Pattenaude, S. R.; Streacker, L. M.; Ben-Amotz, D.; Markland, T. E. The Interplay of Structure and Dynamics in the Raman Spectrum of Liquid Water over the Full Frequency and Temperature Range. *The Journal of Physical Chemistry Letters* **2018**, *9*, 851–857.
